# Mechanical Signaling: Molecular Mechanisms, Biological Functions, Diseases, and Therapeutic Targets

**DOI:** 10.1002/mco2.70523

**Published:** 2025-12-08

**Authors:** Yicen Long, Peng Wang, Jiacheng Lei, Baihai Su, Qiang Wei, Xiaojing Liu

**Affiliations:** ^1^ College of Polymer Science and Engineering National Key Laboratory of Advanced Polymer Materials Sichuan University Chengdu China; ^2^ West China School of Basic Medical Sciences & Forensic Medicine Sichuan University Chengdu China; ^3^ Sauvage Laboratory for Smart Materials School of Materials Science and Engineering Harbin Institute of Technology (Shenzhen) Shenzhen China; ^4^ Department of Nephrology Kidney Research Institute West China Hospital Sichuan University Chengdu China

**Keywords:** mechanobiology, mechanotransduction, cancer, fibrosis, aging

## Abstract

Cell mechanics is a fundamental regulator of numerous cellular processes, orchestrating critical biological activities spanning from embryogenesis to senescence. Cells continuously sense and respond to mechanical cues through specialized interactions between membrane‐bound adhesion proteins, such as integrins, and adhesive ligands within the extracellular matrix (ECM). This bidirectional interaction forms the basis of mechanotransduction—a complex, dynamic process that ultimately leads to alterations in nuclear mechanics and governs essential cellular functions, including migration, tissue morphogenesis, and so on. In this review, we provide an overview of these dynamic cell–ECM interactions and delve into the intricate molecular mechanisms underlying mechanotransduction. We further introduce advanced research methodologies and emerging clinical tools used to investigate cellular mechanical phenotype, mechanotransduction, and diseases progression. In addition, we analyzed the roles of mechanical biomarkers in the development and progression of cancer, fibrosis, and aging. We highlighted the necessity of drug development targeting mechanotransduction, providing examples of drugs that have already entered clinical trials and preclinical tools. By integrating current findings and outlining emerging perspectives, this review aims to provide critical insights and inspire future efforts in understanding, manipulating, and clinically exploiting mechanotransduction‐targeted markers to regulate the progression of diseases such as cancer, fibrosis, and aging.

## Graphical abstract

In this review, we summarize cell–ECM interactions and delve into the complex mechanotransduction. We introduce advanced research methods and emerging clinical tools used to study cellular mechanical phenotypes, mechanotransduction, and disease progression. Additionally, we analyze the role of mechanical biomarkers in the development and progression of cancer, fibrosis, and aging and emphasize the necessity of drug development targeting mechanotransduction.

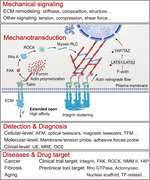

## 1 Introduction

Biomechanics, originating from the Greek words βίος (bios, “life”) and μηχανική (mēchanikē, “mechanics”), is an interdisciplinary field that applies mechanical principles to living organisms, ranging from whole bodies to individual cells [1]. The foundations of biomechanics date back to ancient Greece, where Aristotle first described the body as a mechanical system [1]. Over the centuries, biomechanics evolved with key milestones, including the discovery of cellular adhesion peptides [2] and integrin receptors [3] that mediate cell–extracellular matrix (ECM) interactions. Advancements in the late 20th century, such as Mina Bissell's work on tissue architecture [4] and Donald Ingber's concept of “dynamic reciprocity” [5], revealed how mechanical signals from the ECM regulate cellular behavior by influencing gene expression and cell morphology. Ingber applied the concept of tensegrity from architecture to cell biology and emphasized that the cytoskeleton must rely on intrinsic prestress to maintain shape and respond immediately to external forces [6]. Subsequently, their team further confirmed the relationship between cytoskeletal stiffness, microtubules, and prestress through methods such as magnetic twisting and traction force microscopy (TFM), transforming “prestress” from a structural hypothesis into a measurable mechanical quantity [7, 8]. Since then, a large amount of research on how cells sense mechanical stimuli and convert them into perceivable signals has made mechanobiology an important and influential field.

The cellular microenvironment, especially the ECM, plays a critical role in physiological and pathological processes by providing not only structural support but also dynamic biochemical and mechanical cues [9, 10]. Cells sense these mechanical signals through specialized receptors like integrins and translate them into intracellular responses that regulate proliferation, differentiation, migration, and apoptosis [11, 12]. Unlike biochemical pathways, which rely on biochemical signals, biomechanic pathways rely on mechanical cues such as shear stress, strain, compression, stretching, and tissue stiffness [12]. These mechanical signals are converted into intracellular signaling molecules that affect key processes from embryonic development to tissue homeostasis. Mechanotransduction interrogates the downstream signaling networks that mechanical cues ignite, primarily focusing on signaling molecules that can induce changes in cellular mechanical properties [13]. This also leads to methodological advancements from the molecular to cellular levels [14]. However, based on the existing evidence, it cannot be simply concluded whether upstream mechanoreceptors change their function through force‐induced conformational changes or merely operate as passive biochemical entities. Furthermore, dysregulation of mechanotransduction is implicated in the pathogenesis of numerous diseases, including cancer [15, 16], fibrosis [17, 18], and aging [19, 20]. Beyond dissecting the individual mechanobiology of cancer, fibrosis, and aging, emerging studies are now seeking to explicate the tripartite interplay among different diseases. This growing body of research has been facilitated by the development of advanced measurement technologies.

Given the intricate interconnections among the cellular microenvironment, mechanotransduction, and diseases progression, this review systematically summarizes and discusses four key areas: (1) the composition, structure, and characteristics of the cellular microenvironment; (2) the molecular and cellular mechanisms underlying mechanotransduction; (3) advanced research methodologies and clinically relevant tools for investigating the mechanotransduction and diagnosis; and (4) the pivotal roles of the cellular microenvironment and mechanotransduction in cancer, fibrosis, and aging, and their potential clinical therapy targets. The review follows this logical sequence to provide a coherent and comprehensive understanding, beginning with foundational knowledge and progressing to technical advances and translational insights. By integrating the latest research findings across these interconnected domains, we aim to offer valuable perspectives that will inspire future studies and contribute to the development of novel strategies for diseases diagnosis and therapy.

## 2 Cellular Microenvironment and the Extracellular Matrix

The cellular microenvironment and the interaction between cells and ECM play a fundamental role in cellular mechanics, influencing survival, proliferation, differentiation, and homeostasis. Disruptions in these interactions are closely associated with various diseases.

### 2.1 Cellular Microenvironment

The cellular microenvironment refers to a series of substances and signaling molecules surrounding cells, interacting with cells to collectively form a complex structure. The components of the cellular microenvironment are diverse, including neighboring cells, ECM, soluble factors, as well as physically related forces, sound, light, electric, magnetic fields, and so on [21]. The primary functions of the cellular microenvironment include participation in fundamental physiological processes such as cellular signaling, metabolism, proliferation, differentiation, and apoptosis, while also maintaining the stability and orderliness of tissue structure. The homeostasis of the cellular microenvironment is the basis for sustaining normal cellular life activities, and abnormal changes in microenvironmental components may lead to the occurrence and development of various diseases, such as cancer, cardiovascular diseases, neurodegenerative diseases, and so on [22]. Hence, investigating the functions and regulatory mechanisms within the cellular microenvironment holds paramount importance in comprehending cellular physiological and pathological processes, and in devising innovative therapeutic strategies.

#### 2.1.1 The Cellular Components of the Microenvironment

In the microenvironment for cell survival, different types of cells contain distinct components. For example, the microenvironment of stem cells is primarily composed of the stem cell niche and supporting cells [23]. In some tissues, the microenvironment of undifferentiated precursor cells, such as stem cells, also includes nearby small blood vessels as key components. For tumor cells, they are surrounded by numerous stromal cells, including inflammatory cells, endothelial cells, adipocytes, and tumor‐associated fibroblasts [24]. These stromal cells interact with tumor cells, promoting their growth and migration, and providing the necessary conditions to evade immune system attacks. Dysfunctional stromal cells can further facilitate tumor metastasis and colonization, while certain stromal cells may play a role in antigen presentation [25]. The cellular components in the microenvironment significantly impact maintaining normal cell functions and promoting tumor development.

#### 2.1.2 The Components of the ECM

The ECM is a three dimensional polymer network synthesized and secreted by cells, which is composed of collagen proteins, laminin, elastin, proteoglycans, glycosaminoglycans, and various cell factors, possessing unique biophysical properties that include both solid and liquid characteristics [26, 27, 28]. Due to their viscoelastic nature, they deform in a time‐dependent manner when subjected to force (i.e., exhibiting partial fluidity) and can recover to their initial form after the removal of applied stress. Diseases such as atherosclerosis, deafness, cancer, and developmental disorders are often caused by abnormal physiological responses to external or internal stimuli [29]. Apart from providing physical support essential for cell survival, the ECM also plays a crucial role in providing key biochemical and biomechanical cues for morphogenesis, differentiation, and homeostasis through interactions with cell adhesion molecules, regulatory factors, and connecting proteins.

The molecules constituting the ECM are diverse and can be broadly categorized into three groups: (1) glycosaminoglycans and proteoglycans; (2) collagen and elastin; (3) noncollagenous glycoproteins such as fibronectin (FN) and laminin [30]. The content of ECM in animal tissues varies depending on the tissue type, with epithelial, muscle, brain, and spinal cord tissues having lower ECM content, while connective tissues exhibit the highest ECM content, aligning with their respective functions [31, 32]. Hyaluronic acid, an important glycosaminoglycan, is abundantly secreted by cells during early embryonic development and tissue injury repair, promoting cell migration and proliferation [33]. Insoluble collagen fiber proteins serve as the structural framework of the ECM, containing adhesive peptide sequences like RGD (Arg–Gly–Asp) and GFOGER (Gly–Phe–Pyl–Gly–Glu–Arg) that support cell adhesion, providing physical support for cell growth [34, 35]. Additionally, FN and laminin, besides containing RGD sequences, also include adhesive peptides such as PHSRN (Pro–His–Ser–Arg–Asn) [36], IDAPS (Ile–Asp–Ala–Pro–Ser), REDV (Arg–Glu–Asp–Val) [37], and IKVAV (Ile–Lys–Val–Ala–Val), PDSGR (Pro–Asp–Ser–Gly–Arg), and YIGSR (Tyr–Ile–Gly–Ser–Arg), serving as binding sites for various integrin heterodimers [38].

In addition to structural proteins, soluble factors embedded within the ECM play critical roles in regulating cell adhesion, proliferation, and migration by interacting with cell surface receptors. For instance, transforming growth factor beta (TGF‐β) binds to integrin α_V_ and becomes mechanically activated, mediating both physiological and pathological processes [39]. These include wound healing, systemic sclerosis, idiopathic pulmonary fibrosis, and cancer progression [40, 41, 42].

### 2.2 Cell–Matrix Adhesion

Cell–matrix adhesion is mediated by specific cell adhesion molecules expressed on the cell surface [43]. This adhesion is indispensable throughout the development of animal organisms, including processes such as fertilization, embryonic implantation, morphogenesis, organ formation, and the maintenance of adult structure and function. Cells not only sense and respond to mechanical cues from the ECM but also actively remodel the mechanical properties of the ECM, thereby modulating subsequent biomechanical signaling in both physiological and pathological contexts.

#### 2.2.1 Integrins

Integrins serve as the primary receptors through which cells perceive and respond to mechanical cues in their environment. In mammals, integrins are transmembrane αβ heterodimers composed of 18 α subunits and 8 β subunits, which form 24 distinct heterodimers in humans [44]. Each integrin is structured into extracellular, transmembrane, and cytoplasmic domains, functioning as a critical bridge that links the ECM to the intracellular cytoskeleton [45]. This coupling enables cells to dynamically sense and adapt to their mechanical environment by connecting ECM ligands to the intracellular actin cytoskeleton [46].

Integrins facilitate bidirectional signal transduction across the plasma membrane. In the outside‐in signaling pathway, integrins transmit mechanical cues from ECM ligands into the cell, triggering intracellular signaling pathways. Conversely, in the inside‐out pathway, integrins are activated by intracellular signals, modulating their affinity for ECM ligands and regulating a wide range of physiological functions [47]. The biophysical properties of the ECM [48], including FN stability [49], local stiffness [50], viscoelasticity [51], fiber Orientation [52], stress relaxation [53], and ligand mobility [54], profoundly influence cellular mechanosensing and mechanotransduction through integrin‐mediated interactions. These integrin–ECM interactions not only regulate biomechanical signaling within cells but also play pivotal roles in tissue fibrosis, immune responses, and other processes [55, 56].

#### 2.2.2 Cell–Matrix Interactions

The process of cell–matrix adhesion occurs in distinct stages, each characterized by specific adhesion structures. During the initial phase of adhesion, integrins bind to ECM proteins such as collagen, FN, and laminin, undergoing conformational changes that activate their intracellular domains. This activation initiates intracellular signaling cascades and promotes cytoskeletal remodeling. Mechanical forces further induce conformational changes in integrins, increasing their binding affinity for both ECM ligands and cytoskeletal adaptor proteins. As integrins cluster, cortical actin filaments in lamellipodia polymerize, and cytoplasmic proteins aggregate to form small, transient adhesion structures known as nascent adhesions. These nascent adhesions either disassemble or mature into larger, more stable focal adhesions [57]. As focal adhesions grow in size (ranging from 1 to 5 µm), additional cytoplasmic proteins assemble, coupling the intracellular actin cytoskeleton with integrins' cytoplasmic domains. This linkage enables the transmission of contractile forces generated by the actomyosin cytoskeleton to the ECM, leading to fibrin remodeling within the ECM. Substrate stiffness significantly influences cell–ECM interactions. Cells cultured on softer substrates generate lower traction forces compared with those on stiffer substrates. This demonstrates the concept of “bidirectional reciprocity,” wherein the mechanical properties of the ECM and the contractile forces of the cell reciprocally influence each other, collectively regulating cell behavior and matrix remodeling.

### 2.3 Cell–Cell Adhesion

Cell–cell adhesion refers to the process by which cells recognize and establish stable connections with one another through homotypic adhesion molecules on their surfaces. In multicellular organisms, this process is fundamental for maintaining tissue architecture and stability. Beyond structural roles, cell–cell adhesion facilitates the exchange of information and substances, promoting coordinated tissue development and organismal growth. Mechanically, cell–cell adhesion influences cell morphology, function, positioning, and differentiation within tissues. Alterations in adhesion strength can significantly impact tissue structure and functionality, ultimately affecting overall organismal homeostasis.

#### 2.3.1 Cadherins

Cadherins are a highly conserved family of transmembrane cell–cell adhesion molecules that are essential for embryonic development, tissue formation, and homeostasis in metazoans. These calcium‐dependent adhesion proteins are classified into four main types: classical cadherins, protocadherins, desmosomal cadherins, and atypical cadherins [58]. Vertebrates express over 100 types of cadherins [59], reflecting their diverse and critical roles in cellular adhesion.

Classical cadherins are characterized by their distinct structural domains: an N‐terminal extracellular domain (EC1‐EC5) with five subunits, a single‐pass transmembrane domain, and a highly conserved C‐terminal cytoplasmic tail [58]. The cytoplasmic tail interacts with β‐catenin, which binds to α‐catenin, forming the cadherin–catenin complex that links cadherins to the actin cytoskeleton [60]. Each extracellular subunit of cadherins exhibits relative rigidity and is connected by flexible hinge regions. Upon initiating cell–cell adhesion, cadherin subdomains bind calcium ions near these hinge regions, resulting in rigid, rod‐like extracellular structures with slight curvature that serve as binding interfaces. The N‐terminal domain undergoes a conformational adjustment to interact with cadherins on adjacent cells.

Although the affinity of a single cadherin–cadherin interaction is relatively weak, cadherins cluster on the cell surface to enhance adhesion strength. Within these clusters, the first extracellular domain (EC1) of a cadherin interacts with the second extracellular domain (EC2) of neighboring cadherins through cis‐interactions [61, 62]. These interactions result in a zipper‐like organization of cadherins, where numerous weak individual bonds collectively create strong cell–cell adhesion.

Cadherin‐mediated adhesion can regulate intercellular tension and stability through three mechanisms: *Adhesion tension*: Directly reducing local interface tension via the formation of adhesive bonds. *Cytoskeletal coupling*: Indirectly reducing local interface tension through connections with the actomyosin cytoskeleton. *Mechanical resistance*: Enhancing local interface stability by counteracting applied forces from neighboring cells [63].

Cadherins not only establish mechanical links to the actin cytoskeleton through catenins but also modulate interactions with actin‐associated signaling molecules. Mechanical activation of cadherins triggers β‐catenin recruitment, which subsequently binds α‐catenin and vinculin, activating a RhoA‐dependent signaling cascade that orchestrates cytoskeletal dynamics and reinforces adhesion strength.

#### 2.3.2 Intercellular Interactions

When cells approach each other, actin filament‐driven membrane protrusions initiate contact between cell surfaces. This initial interaction results in the localized clustering of cadherins and catenins, which triggers intracellular signaling pathways. Additional cadherins and catenins are recruited to the contact zone, while the cortical actin network undergoes remodeling, expanding the intercellular contact area. The recruitment of myosin further organizes a contractile actin network that connects to cadherin complexes and tight junctions, enabling the transmission of mechanical stress across cells.

Cadherin‐mediated cell–cell adhesion plays a vital role in maintaining tissue integrity by ensuring mechanical cohesion and facilitating stress distribution. This adhesion mechanism is central to tissue morphogenesis and the preservation of tissue homeostasis [64].

The cellular microenvironment is a dynamic and complex system composed of both cellular components and noncellular elements, primarily the ECM. The ECM provides not only structural scaffolding but also biochemical and biomechanical cues that regulate cellular behavior. Through integrin‐mediated cell–matrix adhesion and cadherin‐dependent cell–cell adhesion, cells continuously sense and respond to their surroundings, maintaining tissue architecture and function. These adhesive interactions are central to mechanotransduction, whereby mechanical signals are converted into biochemical responses, ultimately influencing cell survival, proliferation, differentiation, and homeostasis.

## 3 Mechanotransduction

From simple bacteria and archaea to complex eukaryotic organisms, survival hinges on the ability of living systems to respond to environmental stressors, including diverse mechanical forces [65]. Throughout evolution, nearly all organisms have developed specialized structures adapted to withstand specific mechanical challenges [66]. In multicellular tissues, cells are subjected to a variety of mechanical forces, such as compression, tension, fluid shear stress (FSS), and hydrostatic pressure. These forces play multifaceted roles in tissue formation, development, and homeostasis [67]. The way cells perceive and respond to these mechanical forces is deeply influenced by the physical properties of the cells themselves, their neighboring cells, and the surrounding ECM [68, 69]. Perturbations in this mechanical homeostasis—such as changes in force distribution, material properties, or cellular responses to mechanical cues—are increasingly recognized as critical contributors to the physical basis of various diseases [70]. For instance, shifts in mechanical equilibrium at the cellular or tissue level can drive pathological processes, underscoring the centrality of biomechanics in health and disease.

A comprehensive understanding of the intricate interplay between mechanical signal perception, subsequent mechanotransduction, and the regulation of cell fate within both cellular and noncellular microenvironments is crucial. Advancing this knowledge will not only deepen our understanding of fundamental biological processes but also pave the way for innovative therapeutic strategies targeting mechanical dysfunctions in disease.

### 3.1 Mechanotransduction Mediated by Integrins

**FIGURE 1:**
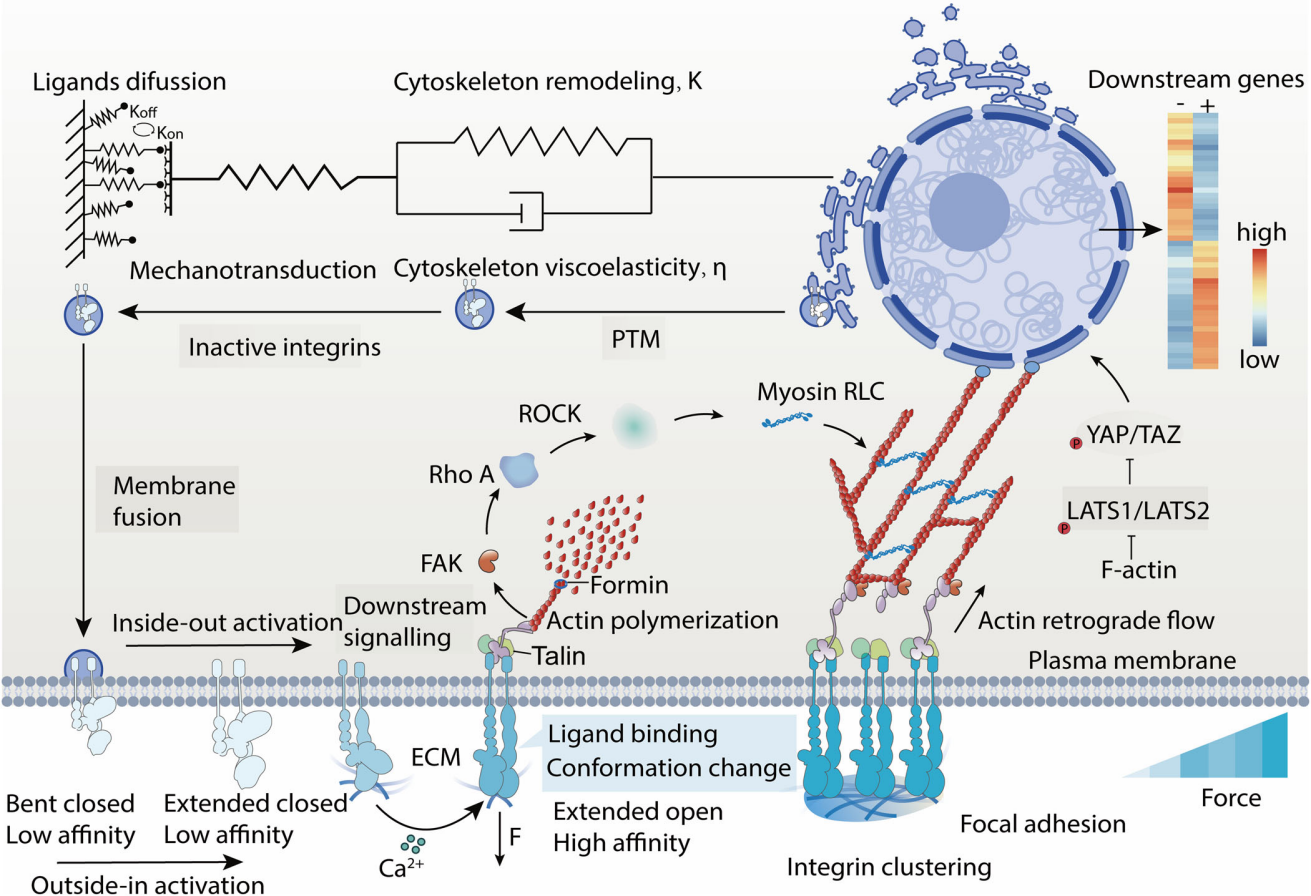
Cellular mechanotransudction from ECM to nucleus. (1) Extracellular ligand binding triggers integrin conformational change, modulating outside‐in force transmission; (2) FA‐related molecules recruit and activate RhoA/ROCK, promoting actomyosin contraction and counterbalancing external force; (3) cytoskeletal visco‐elasticity transmits tension to focal adhesions and the LINC complex, exposing cryptic binding sites and triggering downstream biochemical signaling pathways; (4) nuclear deformation alters chromatin accessibility and gene expression, modulating integrin trafficking. *Abbreviations*: Ca^2^⁺, calcium ion; F, external force; ECM, extracellular matrix; FAK, focal adhesion kinase; Rho A, Ras homolog family member A; ROCK, Rho‐associated kinase; Myosin RLC, myosin regulatory light chain; YAP/TAZ, Yes‐associated protein/transcriptional coactivator with PDZ‐binding motif; LATS1/2, large tumor suppressor kinase 1/2; PTM, posttranslational modification; P, phosphorylated.

As shown in Figure 1, integrin‐mediated sensing of the cellular microenvironment is pivotal for cell adhesion and mechanotransduction. Binding of integrin α and β subunits to extracellular ligands induces conformational changes that activate their intracellular domains, thereby facilitating linkage to actin filaments through associated structural proteins. Contractile forces generated by myosin through ATP hydrolysis along actin filaments are transmitted to the intracellular integrin domains. In concert with ligand–receptor adhesion forces, these mechanical inputs induce conformational alterations in integrins, exposing previously concealed binding sites and initiating downstream biochemical signaling pathways [57, 71].

Under mechanical tension, integrins transition through a series of conformational states—from a “closed conformation” to an “extended‐closed conformation,” and ultimately to an “extended‐open conformation”—leading to an over 1000 fold enhancement in binding affinity [57]. These force‐induced changes within the NPxY motif of the integrin β subunit enable binding to the F3 domain of talin, which undergoes structural extension. This process reveals specific binding sites for a variety of cytoskeletal proteins (e.g., vinculin, paxillin, α‐actinin, zyxin), adaptor proteins (e.g., Kank family, DLC1), and signaling molecules (e.g., FAK, Src, Rho family GTPases, p130Cas) [60, 64, 72]. Notably, talin's rod‐like region contains 9 vinculin‐binding sites among its 13 structural domains [72]. Vinculin interacts with talin through its D1 domain and binds actin filaments or PIP2‐rich membranes via its tail domain [71]. This binding relieves vinculin's autoinhibited conformation, enabling its interaction with additional proteins such as paxillin, the actin‐related protein 2/3 (Arp2/3) complex, tensin, VASP, zyxin, vinexin, ezrin, and FAK. These interactions, facilitated by Rac1 and PI3K, promote the recruitment of the Arp2/3 complex to vinculin‐binding sites, thereby enhancing local actin nucleation, maturation of adhesion plaques, and reinforcement of stress fibers [73, 74].

Interestingly, the transmission of force from the ECM to the nucleus occurs with remarkable speed, despite the viscoelastic nature of the cytoplasm. While small molecules like calcium ions take approximately 25 s to diffuse 50 µm and molecular motors require about 50 s to transport cargo over the same distance, mechanical forces propagate 50 µm along stress fibers in a mere 2 µs [75].

These forces are transmitted to the nucleus via the linker of nucleoskeleton and cytoskeleton (LINC) complex, which physically connects the cytoskeleton to the nuclear envelope. The LINC complex comprises Nesprin (nuclear envelope spectrin‐repeat proteins), SUN (Sad1p, UNC‐84), and nuclear lamina proteins. Nesprin 1 and Nesprin 2, located on the outer nuclear membrane (NM), link actin filaments to SUN1 and SUN2 on the inner NM. Simultaneously, SUN1 interacts with Lamin A and the nuclear pore complex within the nuclear lamina. This coupling establishes a direct physical pathway from the ECM to the nuclear skeleton, providing an efficient mechanism for transmitting extracellular mechanical signals to the nucleus [76].

### 3.2 Biochemical Signaling Transduction Mediated by the FAK–Src Complex

Nonreceptor tyrosine kinases, particularly FAK and Src family kinases, form a critical dual‐kinase complex—referred to as the FAK–Src complex—in integrin‐mediated mechanotransduction. Conformational changes in the cytoplasmic domain of the integrin β subunit trigger autophosphorylation of FAK at tyrosine 397, which creates a high affinity binding site for the Src homology 2 domain of Src [77]. The subsequent binding of Src to FAK induces conformational activation of Src, leading to the formation of a transient FAK–Src signaling complex [78]. Within this complex, Src phosphorylates FAK at tyrosines 576 and 577 in the kinase domain activation loop, as well as at tyrosines 861 and 925 in the C‐terminal domain [79]. These phosphorylation events enhance the kinase activity of the FAK–Src complex and generate additional docking sites for downstream signaling proteins.

The active FAK–Src complex interacts with the proline‐rich (PR1 and PR2) domains of p130Cas via FAK, while also binding p130Cas through the SH3 domain of Src [80, 81]. Additionally, the FAK–Src complex phosphorylates paxillin, thereby facilitating the recruitment of ArfGAP–paxillin–kinase linker (PKL) and guanine nucleotide exchange factors (GEFs), such as Pak‐interacting exchange factor‐beta (β‐PIX) [82].

Through these interactions, the FAK–Src complex coordinates the spatial and functional regulation of GEFs, modulating integrin‐dependent signaling pathways that control key cellular processes, including adhesion, migration, and mechanotransduction, with remarkable specificity and precision.

### 3.3 Rho Family Signaling Cascade

The Rho GTPase family, a key subgroup within the Ras homologous (Rho) protein family (∼21 kDa), plays a central role in cellular mechanotransduction, regulation of the actin cytoskeleton, cell cycle progression, and gene transcription [83]. Like Ras proteins, Rho GTPases function as molecular switches, cycling between distinct conformational states depending on whether they are bound to GDP (inactive) or GTP (active). GTP‐bound Rho proteins actively transduce signals by interacting with downstream effector molecules. Among the 22 identified Rho family members, Rac, cell division cycle 42 (Cdc42), and RhoA are particularly critical in mediating cellular mechanotransduction. The actin cytoskeleton is highly dynamic, with the assembly and disassembly of actin filaments playing essential roles in determining cellular vitality, division, and the formation of specialized structures [84].

**FIGURE 2:**
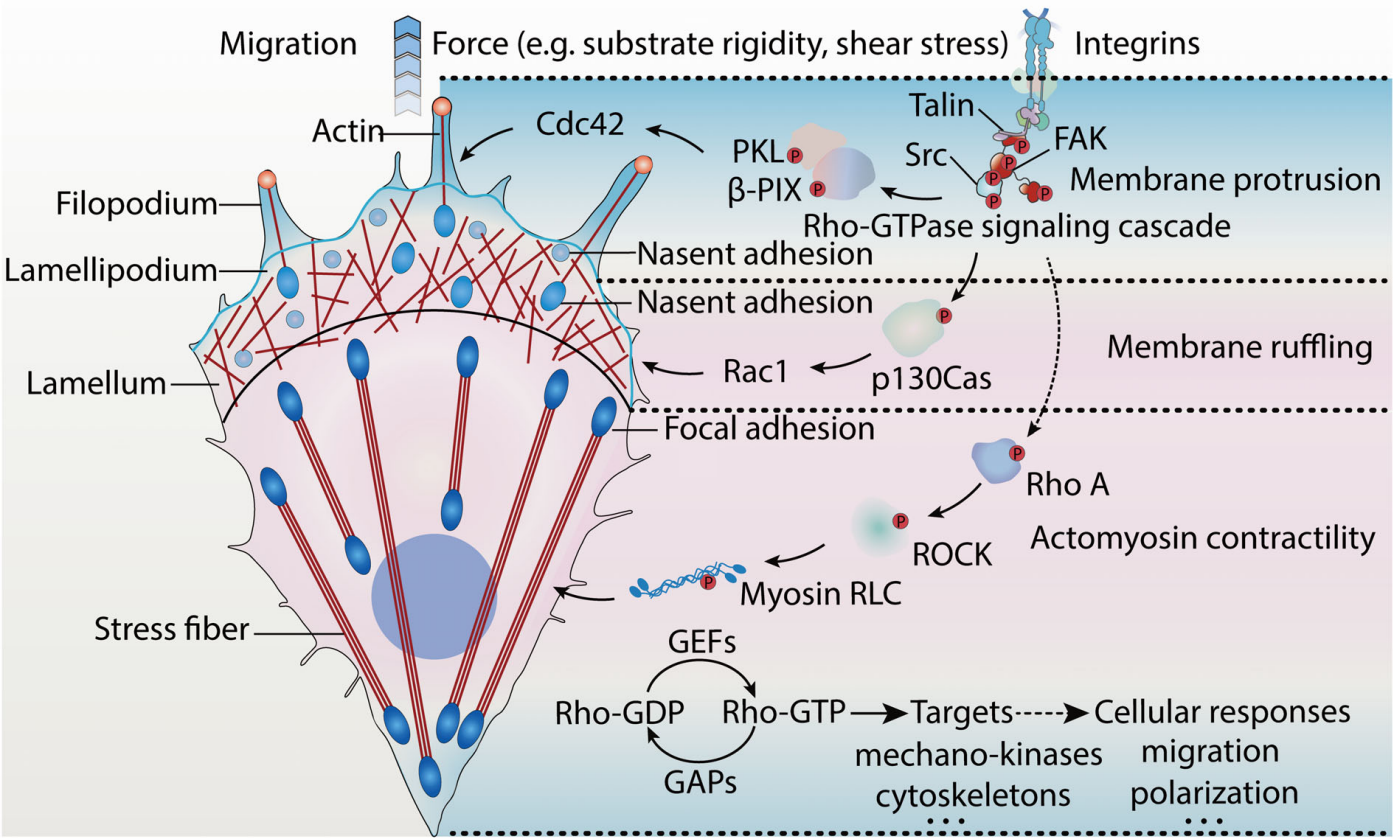
The integrin‐regulated signaling cascade of Rho GTPases. Mechanical cues such as matrix stiffness and shear stress regulate cellular behavior through integrin‐mediated activation of Rho GTPases. Cdc42, modulated by PKL and β‐PIX, plays a critical role in filopodia formation. As adhesions mature into focal adhesions, RhoA promotes the assembly of stress fibers via the RhoA/ROCK pathway, thereby modulating the cellular response to mechanical stimuli. In migrating cells, Rac is activated at the leading edge and facilitates the formation of lamellipodia, contributing to directional movement. *Abbreviations*: Cdc42, cell division cycle 42; PKL, paxillin–kinase linker; β‐PIX, Pak‐interacting exchange factor‐beta; Src, proto‐oncogene tyrosine protein kinase Src; FAK, focal adhesion kinase; RhoA, Ras homolog family member A; ROCK, Rho‐associated kinase; Myosin RLC, myosin regulatory light chain; GEF, guanine nucleotide exchange factors; GAP, GTPase activating protein; Rac1, Rac family small GTPase 1; p130Cas, Crk‐associated substrate; P, phosphorylated.

Figure 2 illustrates the integrin‐regulated Rho GTPase signaling cascade. Rac primarily drives the formation of lamellipodia at the leading edge of migrating cells by regulating the de novo assembly of peripheral actin filaments [85]. The Arp2/3 complex, composed of seven subunits, binds to actin filament sides at the leading edge, playing a key role in actin polymerization [86]. Rac activates the Arp2/3 complex, thereby promoting actin polymerization [87]. Furthermore, activated LIM domain‐containing kinase (LIMK) phosphorylates cofilin at its third serine residue, inactivating cofilin's actin depolymerization activity and increasing the pool of polymerized actin [88]. Rac‐mediated LIMK activation, via protein kinase A, further diminishes cofilin's depolymerizing effect on actin.

Cdc42 is crucial for the regulation of filopodia formation. GTP‐bound Cdc42 directly binds to the N‐terminal regulatory domain of p21‐activated kinase (PAK), thereby activating PAK and promoting actin reorganization [89]. Cdc42 also activates Wiskott–Aldrich syndrome protein (WASP) by relieving its autoinhibitory interaction, allowing WASP to signal through the Arp2/3 complex and regulate the dynamics of the actin network at the leading edge, promoting actin polymerization. Additionally, Cdc42 can inhibit cofilin activity, further suppressing actin depolymerization [90].

RhoA, a key member of the Rho family, is essential for the formation of actin stress fibers and the regulation of focal adhesion signaling. Two critical downstream effectors of RhoA, Rho‐associated kinase (ROCK) and mammalian Diaphanous homolog (mDia), are central to this process. ROCK, the first identified RhoA effector, enhances its kinase activity upon binding to GTP‐bound RhoA [91]. ROCK phosphorylates myosin light chain phosphatase, thereby reducing its activity and promoting myosin light chain phosphorylation, which activates myosin. The primary function of ROCK is to drive the formation of stress fibers and focal adhesions by activating myosin [92].

The small GTPase Rho induces the formation of actin stress fibers and mediates the assembly of different actin structures. RhoA–GTP disrupts the intramolecular interaction of mDia, leading to its activation [93]. Active mDia induces the formation of actin stress fibers, which are disordered in the absence of ROCK activity. Furthermore, active mDia transforms the dense actin fibers induced by ROCK into oriented stress fiber structures. Thus, the interplay between mDia and ROCK downstream of RhoA regulates the assembly of actin stress fibers, contributing to cellular mechanics [94].

The Rho family GTPases control the dynamics of the actin cytoskeleton by cycling between active GTP and inactive GDP states, driving extensive changes in cellular mechanics. While there is functional overlap among the different Rho GTPases, each is specifically activated by distinct environmental signals, which in turn elicit unique changes in the actin cytoskeleton.

### 3.4 Nuclear Mechanotransduction

Nuclear mechanotransduction refers to the process by which mechanical forces are detected and transduced within the cell, ultimately influencing nuclear architecture and function. Although much of the current research in mechanotransduction has focused on the effects of mechanical stimuli on the plasma membrane and cytoplasm, accumulating evidence underscores the critical role of the nucleus in responding to mechanical cues. The nucleus, as the central hub of gene regulation, is highly sensitive to physical forces that can induce changes in its shape, mechanical properties, and chromatin organization, thereby modulating gene expression. These nuclear responses to mechanical stimuli are essential for regulating key cellular processes, including differentiation, migration, and aging, and are fundamental to maintaining tissue homeostasis. In this section, we explore the molecular mechanisms through which mechanical forces are transmitted to the nucleus and how these forces influence chromatin dynamics and gene transcription, ultimately shaping cellular behavior.

Contractile forces applied to the LINC complex are transmitted via Sun proteins, which connect to nuclear lamina proteins and other nuclear scaffolds. This mechanism serves as a conduit for transmitting physical signals from the plasma membrane to the nucleus [76]. Nuclear mechanical properties are determined by the complex interactions between chromatin, the nuclear lamina, and cytoskeletal filaments [95, 96]. Mechanical tension exerted on the nucleus leads to alterations in the phosphorylation dynamics of nuclear lamina proteins, resulting in nuclear pore dilation and changes in nuclear morphology [97]. The opened nuclear pores facilitate the translocation of force‐sensitive transcription regulators, such as Yes‐associated protein (YAP)/ transcriptional coactivator with PDZ‐binding motif (TAZ) [98] and MKL1 [99], from the cytoplasm to the nucleus, thereby assisting in the subsequent expression of target genes. Within the nucleus, chromatin binds to the inner NM through LEM (LAP2, emerin, and MAN1) domain‐containing members, forming lamin‐associated domain (LAD) regions [100]. A defining feature of nuclear mechanotransduction is the redistribution of NM proteins, such as emerin, which undergo turnover in response to mechanical stimuli [101]. Under conditions of low nuclear tension, the cell nucleus exhibits relative softness, and chromatin is more condensed [102]. Highly dense arrays of nuclear bodies induce the heightened activity of methyltransferases, specifically Ezh1/Ezh2 in the Polycomb family PCR2 complex [103], resulting in the methylation of H3K27me3 [104, 105]. This methylation further recruits the PCR1 complex to bind to H3K27me3 sites [103], leading to the localized diffusion of adjacent histones [106, 107]. Histone methylation facilitates the recruitment of large chromatin‐repressive complexes, including histone deacetylases (HDACs), which enhance histone binding to DNA by deacetylating lysine residues, thereby increasing the positive charge of histones and reinforcing their interaction with DNA [108, 109]. The aforementioned series of posttranslational modifications collectively contribute to the dense peripheral chromatin structure [110], creating a challenging environment for proteins responsible for activating various transcription processes to approach. Consequently, this forms gene‐poor heterochromatin regions [105]. When nuclear tension increases, the enlarged nuclear envelope pulls the heterochromatin of LAD regions through LEM family proteins [101]. The resultant forces not only shift nuclear bodies but also lead to the stepwise decondensation/unfolding of chromatin fiber structures [110], disrupting previously stably bound chromatin repression complexes, such as NOCR–SMRT–HDAC [104, 111]. Discrete arrays of nuclear bodies and a loose nuclear structure provide spatial and binding domains for the recruitment and binding of H2B monoubiquitinase Rad6/Brel, histone acetyltransferases [112], TATA‐binding proteins, ATP‐dependent chromatin remodeling complexes [113], and Trithorax family [114]. Additionally, this facilitates the recruitment and binding of RNA polymerase II [115], enabling the transcription of force‐sensitive‐related genes [116, 117].

### 3.5 The Translocation of Force‐Sensitive Transcription‐Related Factors

Cell behavior is intricately regulated by mechanical signals from the cellular microenvironment, where factors such as blood flow, muscle contraction, and tissue rigidity generate macroscopic mechanical stimuli that influence molecular‐level responses within cells. A critical area of investigation has been understanding how these mechanical signals are perceived and transduced at the molecular level to regulate specific gene expression. Recent research has increasingly focused on how force‐sensitive transcription‐related factors function as intracellular mechanoreceptors, mediating the transmission of mechanical signals to the nucleus.

**FIGURE 3:**
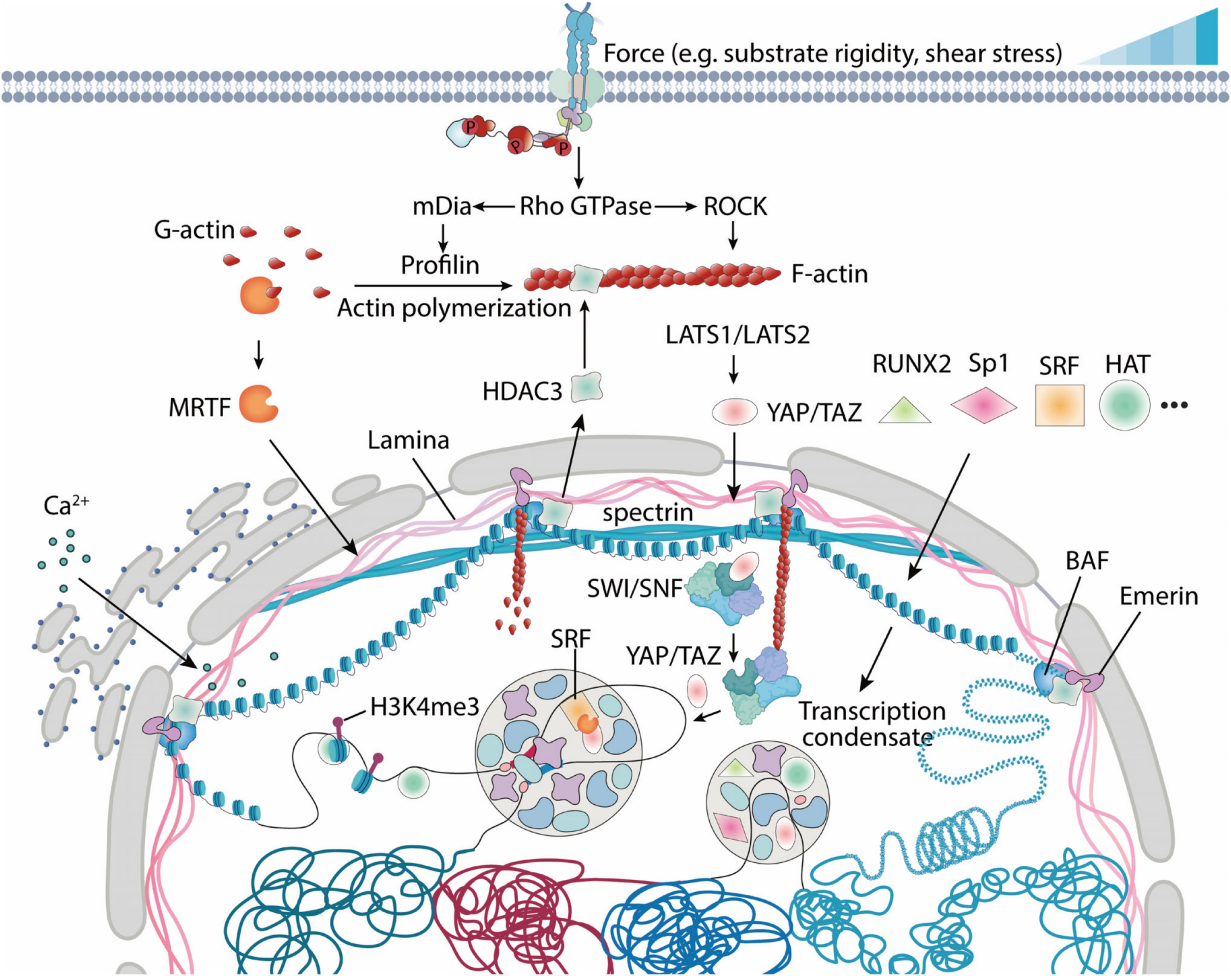
Mechanical cues tune actomyosin stability and transcription‐related factors shuttling. (1) Extracellular stress enhances cytoplasmic F‐actin polymerization, lowering G‐actin and releasing MRTF for nuclear import–SRF‐driven cytoskeletal gene transcription; (2) elevated cytoplasmic tension drives YAP into the nucleus; once inside, abundant nuclear F‐actin polymerizes and captures SWI/SNF, thereby releasing YAP/TAZ to interact with transcription factors such as RUNX2 and modulate gene expression; (3) low nuclear tension permits HDAC3 entry to deacetylate histones, condensing chromatin and silencing genes, whereas high tension stretches LEM‐domain emerin to pull LAD–heterochromatin, mechanically unfolding the fiber and evicting the NCOR–SMRT–HDAC3 complex to enable transcription. *Abbreviations*: mDia, mammalian Diaphanous homolog; ROCK, Rho‐associated kinase; MRTF, myocardin‐related transcription factor; SRF, serum response factor; YAP/TAZ, Yes‐associated protein/transcriptional coactivator with PDZ‐binding motif; SWI/SNF, SWItch/sucrose nonfermentable chromatin‐remodeling complex; BAF, BRG1/BRM‐associated factor; RUNX2, Runt‐related transcription factor 2; LATS1/2, large tumor suppressor kinase 1/2; HDAC3, histone deacetylase 3; HAT, histone acetyltransferase; H3K4me3, trimethylated histone H3 lysine 4; Sp1, specificity protein 1; Ca^2+^, calcium ion.

The identification of YAP and TAZ as mechanosensors has made significant strides in bridging gaps in our understanding of this process. YAP and TAZ, homologs of the Yorkie protein, act as transcriptional coactivators and serve as central nuclear hubs for mechanosignaling triggered by ECM stiffness and actomyosin contractile force [98]. Notably, this regulation is independent of the NF2/Hippo/LATS pathway but requires Rho GTPase activity and tension generated by the actomyosin cytoskeleton [118]. As illustrated in Figure 3, when strong mechanical signals impact the microenvironment, integrin‐mediated cell adhesion is enhanced, promoting mechanotransduction in the cytoplasm. This leads to an increase in tension within the actomyosin cytoskeleton, which subsequently relieves the 14‐3‐3‐mediated cytoplasmic sequestration of YAP. This release allows YAP to translocate into the nucleus, where it forms a complex with TEA domain transcription factor (TEAD) and binds to gene enhancers. Additionally, the YAP/TAZ–TEAD complex interacts with chromatin remodeling factors and modulates RNA polymerase II, driving or inhibiting various cellular processes such as the cell cycle, migration, and cell fate determination. Bioinformatics analysis of the regulatory regions bound by the YAP/TAZ–TEAD complex has revealed interactions with transcription factors such as p73, RUNX, and TBX5, which regulate the expression of relevant target genes [119].

Serum response factor (SRF), a MADS‐box transcription factor, controls the expression of immediate‐early genes regulated by growth factors, including c‐fos, cytoskeletal actin, and muscle‐specific genes [120]. SRF forms regulatory complexes with other transcription factors through its DNA‐binding domain [121]. For instance, at the c‐fos promoter, SRF interacts with ETS domain triple factor family members, enabling transcriptional control via MAP kinase signaling [122]. SRF activity is regulated by Rho GTPase signaling pathways, independent of TCF [123].

MAL (MKL1/2, MRTF1/2), a coactivator of SRF, is involved in this process. Studies have shown that Rho GTPase‐mediated actin signaling regulates the subcellular localization of MAL. In serum‐starved cells, MAL predominantly resides in the cytoplasm, while upon serum stimulation, it accumulates in the nucleus [124]. In the context of Rho GTPase‐induced actin polymerization, MAL translocates from the cytoplasm to the nucleus, as depicted in Figure 3. The expression of inactivated forms or mutations of LIMK, mDia, and VASP, which are key regulators of the cytoskeleton, disrupts serum‐induced SRF activation [125]. These findings underscore how the activation of the Rho family actin signaling pathway regulates the nuclear accumulation of MAL, thereby promoting the expression of SRF target genes. Within the structure of MAL, the N‐terminal sequence contains two RPEL motifs that are responsive to Rho GTPase signals, while other regions mediate its nuclear export (cytoplasmic retention) and nuclear import [126].

Across biological systems, mechanical forces act as fundamental regulators of cellular behavior, influencing processes such as morphogenesis, tissue integrity, and disease progression. Rather than passively enduring these forces, cells are equipped with sophisticated machinery to detect and convert mechanical inputs into biochemical signals, a process known as mechanotransduction. Central to this are integrin‐based adhesion complexes that bridge the ECM and cytoskeleton, initiating signaling cascades such as the FAK–Src complex and Rho GTPase pathways, which govern cytoskeletal dynamics and contractile tension. These mechanical signals are further conveyed to the nucleus via the LINC complex, impacting gene expression through alterations in chromatin structure or the localization of mechanosensitive transcription‐related factors like YAP/TAZ and MAL. Together, these interconnected pathways form a dynamic network that enables cells to interpret and adapt to mechanical cues. To gain a more quantitative and functional understanding of such complex mechanobiological processes, it is essential to employ biomechanical measurement techniques that can precisely probe force generation, transmission, and cellular responses.

## 4 Biomechanical Measurement Techniques

From the human body to individual cells, biological systems at various scales need to respond to mechanical signals. At the cellular level, precise measurement and recording of specific mechanical signals are crucial for a deeper understanding of cellular behavior and disease mechanisms.

### 4.1 Cellular Level Techniques

**FIGURE 4:**
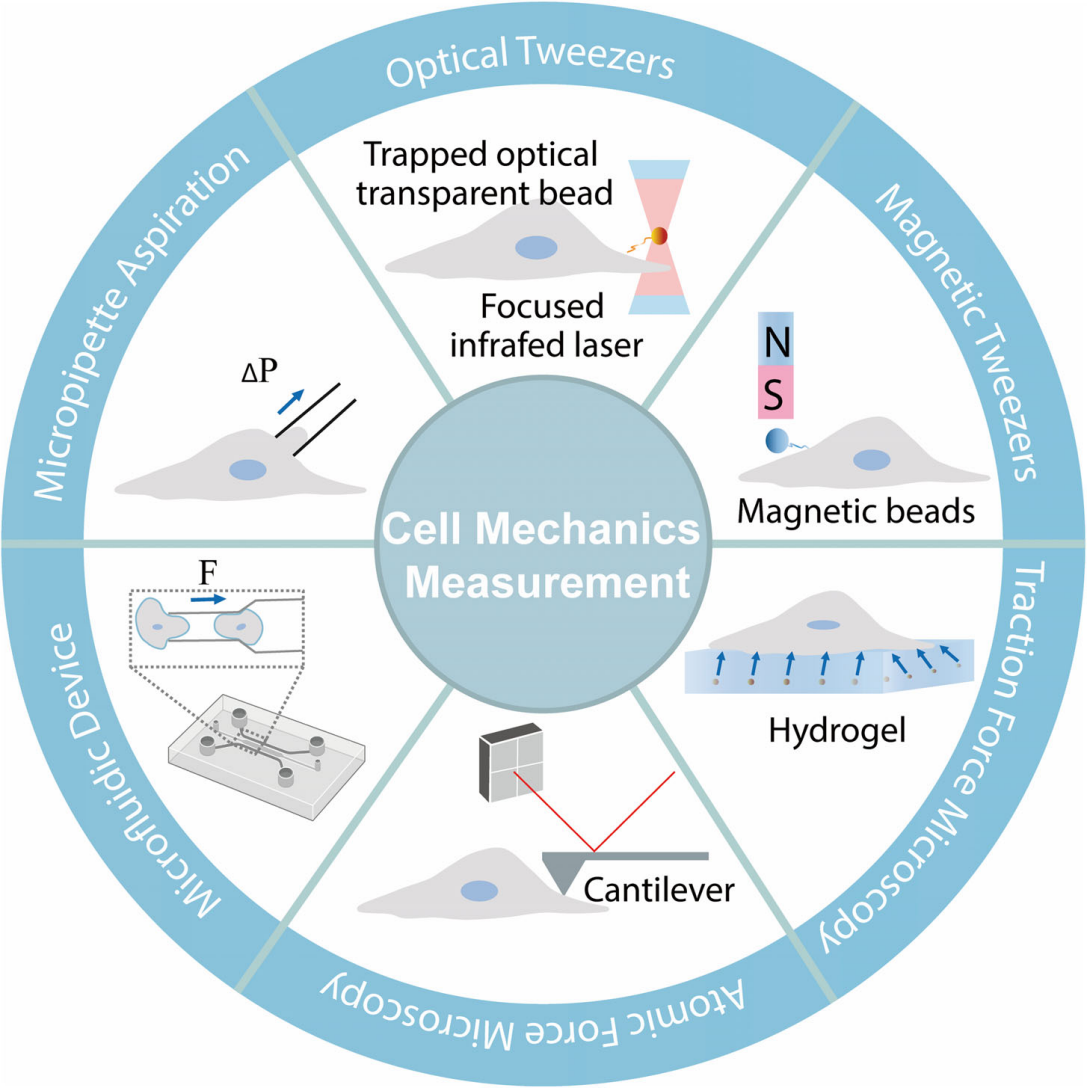
Toolbox for quantifying cellular level forces and mechanics. (1) Atomic force microscopy: cantilever probe indents cell surface, then laser deflection from cantilever bending infers mechanical properties; (2) optical tweezers: highly focused laser beam forms optical trap, applies forces to manipulate cells or microspheres; (3) magnetic tweezers: controlled magnetic field acts on magnetic beads for real‐time measurement of single‐molecule or cytoskeletal mechanical responses; (4) traction force microscopy: tracks displacement of embedded fluorescent microbeads to measure substrate deformation caused by cells on elastic surface and computationally reconstructs traction force. (5) Micropipette aspiration: applies negative pressure to aspirate part of cell membrane into glass pipette and aspirated length derives membrane tension and viscoelastic characteristics; (6) Microfluidic device: applies shear stress or compression in precisely designed channels and analyzes cell deformation under these mechanical stimuli to quantify cell stiffness.

In recent years, with the continuous development of biomechanical measurement techniques, such as atomic force microscopy (AFM), optical tweezers, magnetic tweezers, TFM, micropipette aspiration, and microfluidics in Figure 4, researchers have been able to decode the mechanical mechanisms of cell–ECM and cell–cell interactions at a more detailed level. These techniques not only provide new insights into the complex processes of diseases initiation and progression but also offer powerful tools for understanding cellular behavior within the related microenvironment. Through these advanced biomechanical measurement methods, we are expected to better meet clinical needs and provide new strategies for the early diagnosis and precision treatment.

#### 4.1.1 Atomic Force Microscopy

AFM is a scanning probe microscopy technique that has emerged as a powerful tool for characterizing the morphological and mechanical properties of biological systems. Capable of measuring various mechanical properties such as elasticity, viscosity, and tension, AFM can also provide detailed topographical features of sample surfaces. Over the past decades, AFM has become an essential platform for the morphological and mechanical characterization of biological systems.

The AFM cantilever deflects when it interacts with a tissue specimen through attractive/repulsive forces or upon contact. A four‐quadrant photodetector records the cantilever deflection by detecting the displacement of a laser beam reflected from the cantilever. These deflection data are then converted into topographical mapping data and, after subsequent processing, can be transformed into various types of force maps representing different mechanical properties [127].

To optimize imaging resolution and obtain accurate mechanical information, it is crucial to select cantilevers with appropriate shapes, stiffness, resonance frequencies, and spring constants based on the specific biological samples being studied. Additionally, when evaluating particular mechanical properties, suitable mechanical models must be chosen for fitting and analysis. Given that different regions of a sample surface may exhibit varying mechanical properties, a large number of force curves obtained from surface measurements are required. This necessity has led to the development of force‐volume mapping and peak force tapping techniques [128].

Since the pioneering work of Cross et al. [129] in 2007, who performed ex vivo mechanical analysis of cancer cells using AFM, a substantial amount of AFM data related to cancer has been accumulated. For instance, convolutional neural networks are utilized to analyze AFM data and revealed the stiffness heterogeneity in breast cancer tissues [130]. Additionally, AFM is used to explore the surface characteristics of cancer cells [131]. In mechanistic studies, the combination of confocal fluorescence microscopy and AFM has shown that cells significantly stiffen when invading a collagen I matrix, a process that is dependent on the Rho–ROCK pathway [132]. To enhance the sensitivity of AFM for biomolecular detection and achieve early cancer diagnosis, surface patterning techniques is employed [133]. Collectively, these studies highlight the significant role of AFM in cancer cell characterization, mechanistic investigation, and diagnostic applications.

AFM has become a pivotal tool in dissecting the mechanobiology of fibrosis. By combining AFM microindentation of intact tissue with single‐fiber nanoindentation, the hierarchical mechanical gradient of human fibrotic lung from the macroscopic scale down to the nanometer regime has been mapped [134]. In cardiac fibrosis, AFM provides nanomechanical information that conventional histological staining cannot reveal, allowing sensitive detection of early or clinically hidden fibrotic regions and responding to the accumulation of noncollagenous ECM proteins and proteoglycans [135]. For connective‐tissue disorders, AFM coupled with two‐dimensional fast‐Fourier transform analysis enables simultaneous nanoscale quantification of collagen fibril distinctness, d‐periodicity, orientation, and linearity [136].

During cellular aging, cell‐intrinsic stiffness undergoes deterministic remodeling. In epithelia, the aging‐dependent loss of tissue resilience is mirrored at the single‐cell level. Epithelial cells that have undergone consecutive passages and become “aged” show an increase in apparent Young's modulus compared with early‐passage cells, and this increase in stiffness quantitatively corresponds with an elevation in intracellular cytoskeletal fibers [137]. Analogously, single left‐ventricular cardiomyocytes isolated from aged rats exhibit a significant rise in apparent elastic modulus compared with young controls, implying that cardiomyocyte‐autonomous stiffening may contribute to the impaired diastolic relaxation characteristic of the senescent heart [138]. High‐resolution AFM topographs of the Caenorhabditis elegans cuticle further resolve biomechanical aging in vivo. Nanomechanical mapping reveals that distinct dietary restriction regimens and longevity‐extending genetic pathways produce divergent healthspan outcomes, demonstrating that AFM can decouple lifespan from health‐span through stiffness‐based biomarkers of cuticular aging [139].

#### 4.1.2 Optical Tweezers

Optical tweezers, a groundbreaking technique first realized by Arthur Ashkin in 1986, have emerged as a powerful tool for manipulating and studying biological samples with high precision [140]. In 1987, Ashkin demonstrated the noninvasive nature of optical tweezers through trapping experiments on various biological samples, highlighting its potential for biological and biomedical applications [141].

The fundamental principle of optical tweezers is based on the conservation of momentum of light. When light interacts with a particle, the particle alters the distribution of the light field, resulting in a change in the light's momentum. This change in momentum is transferred to the particle, effectively trapping it. By manipulating the direction and intensity of the light beam, researchers can precisely control the movement of the particle, enabling a wide range of experimental possibilities [142].

In cancer research, optical tweezers have found extensive applications [143]. For instance, they have been used to directly capture lymphocytes [144] and assess the differentiation state of cancer cells by measuring the shortest time required for cell adhesion to occur [145]. Optical tweezers have also been combined with Raman spectroscopy to analyze cancer cells [145] and been used to screen for cancer biomarkers [146]. Additionally, they have been employed to measure the mechanical properties of cell membranes [147] and investigate the relationship between protein folding and cancer development [148].

Using a laser–tweezers–micropillar platform, individual FN fibrils extracted from fibrotic matrices were subjected to cyclic tensile tests. The majority of fibrils exhibited strain hardening, and virtually all displayed an initial stress‐relaxation phase followed by an inverse stress‐relaxation event, thereby furnishing a quantitative mechanical–biological framework for decoding mechanotransduction in both development and fibrosis [149].

In parallel, laser optical tweezers were deployed to interrogate the viscoelastic signature of single dermal fibroblasts isolated from 14 human donors aged 27–80 years. Cell‐stiffness tomography revealed a significant aging‐dependent increase in apparent Young's modulus, indicating that intrinsic stiffening of dermal fibroblasts is a biomechanical hallmark of human skin aging in vivo [150].

#### 4.1.3 Magnetic Tweezers

Magnetic tweezers, initially employed by Crick and Hughes to study cytoplasmic viscoelasticity [151], have since been widely applied in cell mechanics and single‐molecule biophysics. This technique utilizes a gradient magnetic field to exert controllable forces on paramagnetic beads [152]. The elongation changes of the connected molecules are then measured through image analysis under a microscope [152]. Magnetic tweezers are capable of conducting long‐term experiments within a force range of 10 fN to 1 nN, with a temporal resolution of 1–10 ms and a spatial resolution at the subnanometer level [153]. Moreover, the control system of magnetic tweezers is independent of the optical imaging system, offering greater stability compared with AFM and optical tweezers [153].

Superparamagnetic iron oxide nanoparticles (SPIO/SPIONs) have been extensively used for targeted drug delivery to tumor sites [154, 155]. The delivery methods include local injection [156], external magnetic field‐guided delivery [157], and coating the nanoparticles with tumor‐specific antibodies [158] for targeted delivery. Additionally, magnetically driven nanomotors have been shown to selectively adhere to the ECM surrounding cancer cells in a charge‐dependent manner [159], highlighting the significant role of the tumor microenvironment (TME) in cancer progression.

Through spontaneous nanoscale tracer motions and magnetic twisting cytometry, single primary vascular smooth muscle cells (VSMCs) isolated from aged rat thoracic aorta were examined, revealing a sustained elevation of actomyosin tension. This stiffening phenotype remained stable under prolonged culture and across a physiologic range of substrate stiffnesses, indicating that elevated cytoskeletal prestress is an autonomous and irreversible biomechanical signature of VSMC aging [160].

#### 4.1.4 Traction Force Microscopy

TFM was first introduced in 1980 by Harris et al. [161], who qualitatively estimated the magnitude of traction forces by observing the deformation of silicone rubber substrates under a microscope. Since then, TFM has evolved into two major categories: pillar‐based TFM systems and bead‐based TFM systems [162]. The fundamental principle of TFM involves cells exerting traction forces on the ECM through adhesion sites, causing substrate deformation. By measuring the displacement of embedded fluorescent beads or the deformation of the substrate and combining this with a mechanical model of the substrate, TFM can quantify the forces exerted by cells.

TFM has found extensive applications in diseases research. It has been used to distinguish between benign and malignant cancer cells based on the magnitude of traction forces [163]. Additionally, TFM has been employed to study the balance between cancer cell adhesion and migration [164], assess collective behavior of cancer cells [165], and evaluate the interactions between cancer‐associated fibroblasts (CAFs) [166, 167] and cancer cells [168]. TFM has also been utilized to investigate the crosstalk between ECM properties and cancer cell behavior [169, 170, 171, 172, 173, 174], providing valuable insights into the mechanobiology of cancer progression.

Also, polyacrylamine‐based TFM has been employed to quantify the aging‐dependence of cellular contractility in endothelial cells [175], fibroblasts [176], and VSMCs [177]. When cultured on identical elastic substrates, cells derived from aged donors generate significantly higher traction stresses than their young counterparts, establishing increased traction force magnitude as a conserved biomechanical marker of cellular aging across multiple vascular and dermal lineages.

#### 4.1.5 Micropipette Aspiration

Micropipette aspiration, first developed in the 1960s by Rand and Burton for measuring the deformability of red blood cells [178], has become a widely used technique in cell mechanics, cell–cell interactions, molecular interactions, and disease‐related studies. This technique applies precise negative pressure through a micropipette to aspirate cells partially or completely, allowing for the measurement of cellular deformation and mechanical properties. By combining the applied pressure, aspiration depth, and micropipette diameter with appropriate mechanical models, micropipette aspiration can quantify mechanical parameters such as the elastic modulus and viscosity of cells [179].

In cancer research, micropipette aspiration has been employed to measure the adhesion [180] and mechanical properties of cancer cells [181, 182, 183]. Additionally, it has been integrated with microfluidic devices to enable rapid mechanical phenotyping of single cells, providing high‐resolution observations [184, 185] and quantification of nuclear deformation within intact cells [186]. These applications highlight the versatility of micropipette aspiration in studying cancer cell behavior and mechanics.

Micropipette aspiration of freshly isolated chondrocytes from healthy human donors revealed a monotonic aging‐dependent increase in equilibrium modulus, instantaneous modulus and apparent cytoplasmic viscosity, indicating that cell‐autonomous stiffening precedes and may precipitate the mechanical failure of aging articular cartilage by impairing mechanotransduction [187]. Comparative aspiration of neonatal versus adult erythrocytes showed that, although neonatal membranes are softer and more extensible, their critical lytic tension is significantly lower [188]. Analogous studies on mouse T lymphocytes identified a passive deformability migration axis as a key biomechanical signature of immunosenescence [189].

#### 4.1.6 Microfluidics

Microfluidics is a technology that leverages the unique fluid dynamics at the microscale, where precise control of small volumes of fluids enables a wide range of applications. At the microscale, the low Reynolds number (typically much less than 1) indicates that viscous forces dominate over inertial forces, resulting in laminar flow. Surface tension and capillary forces can drive fluid motion passively without the need for external pumps. Furthermore, designing complex geometric structures or using external fields (such as force fields, acoustic fields, or electric fields) can enhance the fluid motion [190, 191].

Microfluidics has been extensively applied in cancer research [192], including studies of tumor–ECM interactions [193], anticancer drug screening [194, 195], cancer cell migration [196], and interactions between cancer cells and immune cells [197, 198]. In clinical diagnostics, microfluidics has shown great promise, particularly in the isolation of circulating tumor cells (CTCs) [199]. The first application of microfluidics for CTC isolation was reported in 2007 [200], with subsequent improvements in automation [201]. Initial approaches relied on physical separation using micropillars, followed by magnetic bead‐based separation targeting surface biomarkers such as EpCAM [202], nucleic acid [203], or using biotinylated antibodies [204]. More recent advancements have focused on improving CTC viability through device optimization [205].

In aging research, microfluidic organ‐on‐chip platforms that recreate a three‐dimensional human microenvironment now permit real‐time tracking of senescence‐associated biomarkers, high‐content screening of prolongevity compounds, and precision elimination of senescent cells. By integrating these functions into a single, miniaturized circuit, the technology offers a high‐throughput, low‐cost bridge between animal models and clinical translation, thereby accelerating the development of antisenescence therapeutics [206].

Currently, mainstream technologies such as AFM, optical tweezers, magnetic tweezers, TFM, micropipette aspiration, and microfluidics have been widely applied in the field of biomechanics, yielding a substantial amount of experimental data. Given the diverse capabilities and limitations of each technique, researchers must carefully weigh the pros and cons based on their laboratory conditions to select the most appropriate method for their specific research needs. Beyond their original use for direct mechanical phenotyping of cancer and aged cells, cell‐level probes can be integrated with biochemical staining and high‐resolution image analysis to quantify ECM stiffness in fibrotic models. Nevertheless, elucidating the molecular origins of the observed changes in cellular and cytoplasmic mechanics still requires complementary molecular‐level tools, including CRISPR‐engineered cytoskeletal mutants, Förster resonance energy transfer (FRET)‐based tension sensors, and mass‐spectrometric mapping of collagen cross‐links, to establish causal links between specific biochemical alterations and emergent biomechanical phenotypes.

### 4.2 Molecular Level Techniques

**FIGURE 5:**
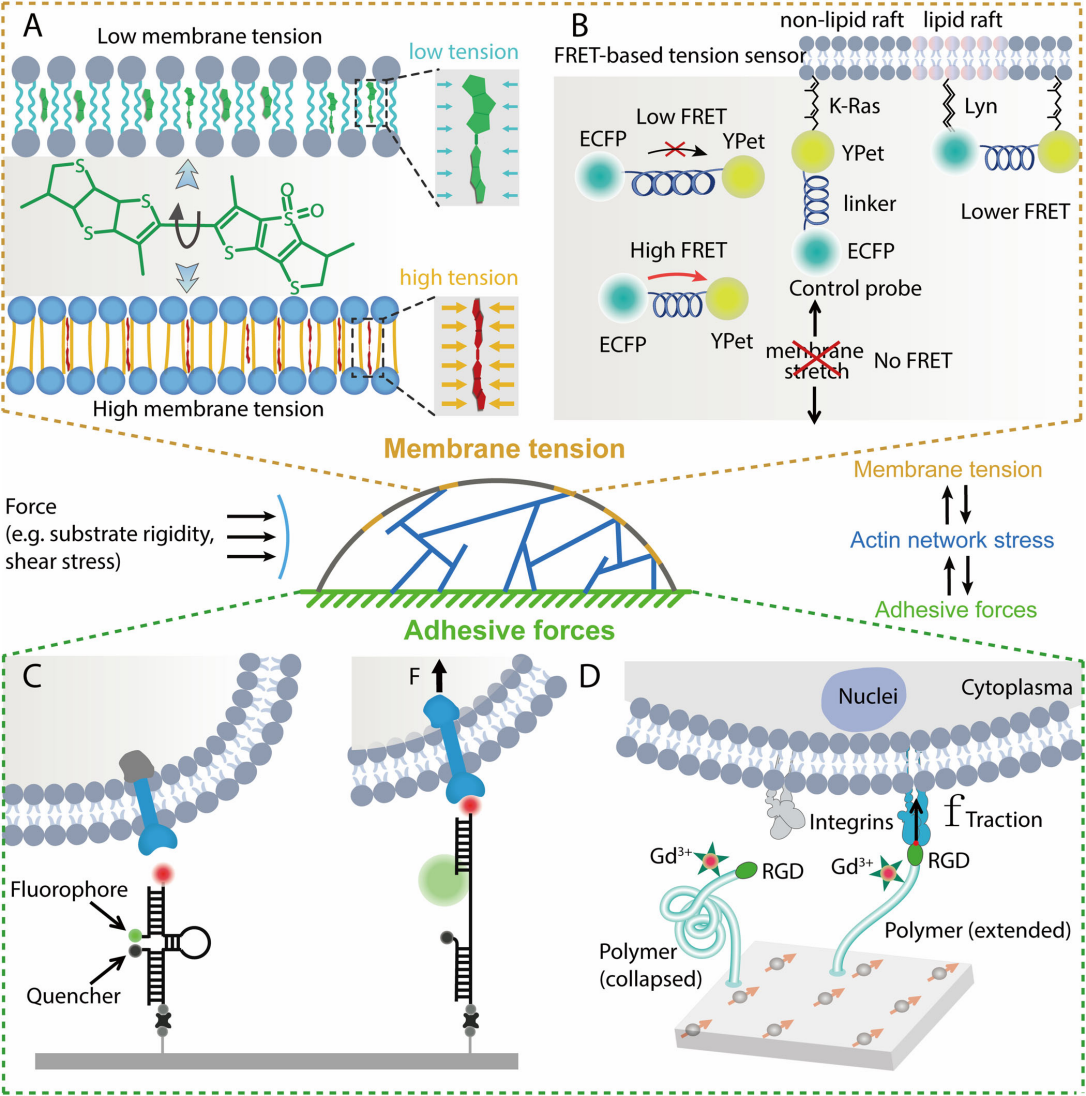
Membrane tension probe and adhesive forces probe. (1) Flipper‐TR: lateral pressure in the lipid bilayer rotates the two dithienothiophene flippers, changing their dihedral angle and polarization; the resultant lifetime shift reports membrane tension; (2) membrane stretching sensor: Lyn (raft) and K‐Ras (nonraft) anchor an elastic linker; membrane stretch elongates it, increasing ECFP–YPet separation and decreasing FRET for reversible tension read‐out; (3) DNA‐based ForceChrono probe: when the mechanical force generated by cell adhesion exceeds the threshold of the probe, the hairpin unfolds, separating the fluorophore and the quencher and restoring fluorescence. After the force is released, the hairpin refolds, enabling reversible and real‐time imaging of cell adhesion forces; (4) quantum‐enhanced diamond molecular tension microscopy: cell‐generated stretch of a force‐responsive polymer alters the distance between PEG‐linked Gd^3+^ and diamond‐NV, modulating *T*_1_ spin relaxation; the measured *T*_1_ change yields pN–nN adhesion forces without fluorescence. *Abbreviations*: FRET, Förster resonance energy transfer; K‐Ras, small GTPase of the Ras superfamily; Lyn, Src family tyrosine kinase; ECFP, enhanced cyan fluorescent protein; YPet, yellow fluorescent protein for energy transfer; RGD, Arg–Gly–Asp adhesion peptide; Gd^3+^, gadolinium ion.

Beyond the cellular level, molecular biology tools mentioned in Figure 5 have also been increasingly utilized in biomechanics research. Tools exploiting conformational rearrangement, linker cleavage, and disruption of weak noncovalent interactions enable quantification of membrane tension and adhesive forces. These tools offer unique insights into the mechanobiology of cellular processes, complementing the information obtained from cellular‐level studies.

#### 4.2.1 Förster Resonance Energy Transfer

FRET is a phenomenon where nonradiative energy transfer occurs between two fluorophores through dipole‐dipole coupling. The donor fluorophore transfers its emitted energy to the acceptor fluorophore, which then emits fluorescence. Typically, the donor and acceptor fluorophores are positioned approximately 1–10 nm apart [207]. FRET can be classified based on changes in donor–acceptor distance or the characteristics of fluorescence. Distance‐based FRET includes cleavage‐based FRET, conformational change‐based FRET, and mechanically induced FRET. Fluorescence‐based FRET is categorized according to the sensitivity of fluorophores to specific conditions, such as pH‐sensitive FRET [208].

In cancer research, FRET has been employed to study the conformation of FN in breast cancer, which may promote tumor vascularization and growth [209]. Additionally, high‐affinity binding peptides have been conjugated to FRET substrates to specifically target enzymes such as MT1–matrix metalloproteinase (MMP). By leveraging the specific binding properties of these peptides, FRET substrates can be localized near MT1–MMP, enhancing the specificity of FRET‐based imaging for this enzyme in tumors [210].

#### 4.2.2 Membrane Tension Probes

Membrane tension probes are essential tools for elucidating the mechanical properties and physiological functions of cell membranes. The Flipper‐TR probe, for instance, exhibits fluorescence lifetime changes that are dependent on the degree of twist between fluorophores. When membrane tension increases, lipid molecules become more ordered, leading to an increase in Flipper‐TR fluorescence lifetime. Conversely, when membrane tension decreases, lipid molecules become more disordered, resulting in a shorter fluorescence lifetime. Flipper‐TR can rapidly label cell membranes in HeLa and MDCK cells, and its fluorescence lifetime changes in response to osmotic stress, making it a valuable tool for visualizing cancer cell migration, spreading, phagocytosis, and detachment [211].

The membrane stretching sensor (MSS) probe is based on FRET and comprises a tension‐sensing module flanked by two anchoring proteins. The tension‐sensing module consists of an elastic spider silk protein inserted between two fluorescent proteins (enhanced cyan fluorescent protein, ECFP, and yellow fluorescent protein, YPet). The probe is anchored to lipid raft and nonraft regions via Lyn and K‐Ras kinases, respectively. Changes in membrane tension alter the distance between the donor and acceptor fluorophores, thereby modulating FRET efficiency. Increased membrane tension stretches the elastic spider silk protein, increasing the distance between the fluorophores and reducing FRET efficiency, while decreased tension enhances FRET efficiency. Bo Liu's team used the MSS probe to investigate membrane tension in HeLa cells under different shear stresses. They found that membrane tension is nonuniformly distributed, with higher tension in the central region and lower tension in the upstream and downstream regions. Membrane tension does not increase proportionally with shear stress but is influenced by membrane fluidity and cytoskeletal integrity. Specifically, increased membrane fluidity and disruption of the actin cytoskeleton lead to higher membrane tension without inducing polarity differences between the upstream and downstream regions [212].

#### 4.2.3 Adhesive Forces Probes

Molecular tension probes are categorized based on their reversibility into tension gauge tether (TGT) and molecular tension fluorescence microscopy (MTFM). TGT detects whether cellular forces irreversibly break double‐stranded DNA, indicating if the mechanical force exceeds a threshold [213]. MTFM, in contrast, utilizes molecular springs that reversibly stretch under cellular forces, quantifying mechanical forces through changes in fluorescence intensity [214]. TGT can detect forces of ∼12 pN with a duration of about 2 s, enabling single‐molecule imaging in live cells and capturing mechanical events with lifetimes shorter than the fluorescence acquisition time [215, 216].

To enhance the sensitivity of probes for weaker and more transient forces, Khalid Salaita's team made several improvements to MTFM. They used DNA hairpins as reversible digital switches to detect transient piconewton forces exerted by T‐cell receptors (TCRs). By incorporating locked oligonucleotides to prevent refolding of stretched DNA probes and unlocked oligonucleotides to trigger toehold‐mediated strand displacement reactions, they achieved information storage and erasure. They optimized the locked nucleotide length to 17mer and adjusted the mechanical threshold (F1/2) to 4.7 pN for TCR force detection, adding Atto647N to the locked oligonucleotide for reporting accumulated mechanical events. These advancements provide insights into how T cells recognize tumor cells of varying stiffness through TCRs [217].

The MCATS technology integrates the CRISPR–Cas12a system with TGT to amplify piconewton‐level force signals. Using DNA probes with ligand‐modified activation strands (e.g., cRGDfK) that bind to cell surface receptors (e.g., integrins), the probes are designed such that the activation strand is hidden until cellular traction forces exceed the mechanical tolerance of the DNA duplex. Upon force‐induced exposure, the activation strand binds to the Cas12a–gRNA complex, activating Cas12a's nuclease activity, which cleaves a fluorescent reporter DNA to produce a detectable signal. The high catalytic activity of Cas12a generates a significant fluorescent signal, detectable by standard fluorescence plate readers [218].

The DNA‐based ForceChrono probe, based on MTFM, employs a dual‐DNA hairpin structure to measure forces at different thresholds and enhances signal sensitivity through fluorescence quenching and enhancement mechanisms. This allows precise measurement of force magnitude, duration, and loading rates at the single‐molecule level in live cells. Zheng Liu's team calibrated these probes using single‐molecule magnetic tweezers, ensuring accuracy across different temperatures and loading rates. Using total internal reflection fluorescence microscopy, they captured real‐time dynamics of single‐molecule forces, revealing nonlinear changes in force duration and loading rates during integrin‐mediated cell spreading. They also demonstrated that focal adhesion proteins (e.g., vinculin) and ligand spacing significantly influence integrin force dynamics [219].

Moreover, quantum measurement technologies have also achieved significant breakthroughs in exploring microscopic forces within cells. Quantum‐enhanced diamond molecular tension microscopy (QDMTM) integrates cell force‐induced stretching of polymers with nitrogen‐vacancy (NV) center spin relaxation time (*T*_1_) measurements. By detecting variations in NV spin relaxation time caused by randomly fluctuating magnetic noise, the adhesive forces exerted by cells on the polymer substrate can be accurately determined. The force‐responsive polymer primarily consists of polyethylene glycol (PEG) chains, which function as molecular springs. Additionally, it incorporates an integrin ligand, Cyclo(RGDfK), and a paramagnetic Gd^3+^ ion. One end of the PEG chain is conjugated to the Gd^3+^ complex, while the other end is anchored to the diamond surface through a thin “active” hybrid silica layer. Stretching or collapsing of the polymer alters the distance between the NV center and the Gd^3+^ ion, thereby influencing the NV spin relaxation time. This enables label‐free measurement of cellular adhesion forces [220]. Compared with conventional fluorescence‐based force measurement methods, QDMTM leverages the quantum properties of NV center electron spins in diamond, offering unprecedented sensitivity and precision. Furthermore, the diamond substrate can be cleaned and reused, enhancing the absolute accuracy for comparative analysis of cellular adhesion forces across different samples.

Although molecular‐level assays have generally matured within cancer research, their deployment in aging studies remains disproportionately scarce. The principal bottleneck is not technical feasibility, but the absence of a systematic annotation linking aging‐related mechanical traits to canonical senescence hallmarks. Quantitative correlations among SA‐β‐gal activity, p16^INK4a^ expression, the senescence‐associated secretory phenotype (SASP) and single‐cell stiffness are still fragmentary, and no “aging‐mechanical fingerprint”—analogous to the universally recognized stiffening of tumor stroma—has yet been identified or therapeutically exploited. By exploiting the high‐resolution mechanical toolkits described above to chart, at single‐cell and single‐molecule resolution, how chromatin stiffness, plasma–membrane tension, and adhesion‐based force transmission evolve with age, we can populate the current mechano‐phenotypic void in cellular senescence. Such a mechanically resolved aging atlas will illuminate druggable mechano‐checkpoints, providing the theoretical bridge necessary to conceive interventions that restore soft‐to‐rigid homeostasis and inaugurate a new class of antiaging therapeutics.

### 4.3 Clinical Level Techniques

Beyond the development of cellular and molecular biomechanical tools, significant progress has also been made in the clinical application of biopsy techniques for diagnostic purposes. Techniques such as ultrasonic elastography (UE), magnetic resonance elastography (MRE), and optical coherence elastography (OCE) have emerged as powerful tools in this domain. Compared with traditional macroscopic measurements like blood pressure and intraocular pressure, these advanced imaging modalities offer broader measurement ranges, superior resolution, and enhanced quantitative capabilities.

#### 4.3.1 Ultrasonic Elastography

UE was developed to overcome the limitations of subjective manual palpation, providing quantitative information on tissue elasticity. UE can detect deeper lesions and superficial masses, offering valuable diagnostic information. Based on its principles, UE is divided into strain elastography (SE) and shear wave elastography (SWE).

SE measures tissue strain by applying stress along the direction of the ultrasound beam. The phase difference in ultrasound signals before and after tissue deformation is converted into displacement, from which strain is calculated. The results are then color‐coded to visually represent tissue stiffness. However, SE results cannot be directly compared across different samples and must be interpreted using metrics such as elasticity scores, strain ratios, and strain size ratios [221].

SE is widely used for grading the malignancy of breast cancers [222, 223, 224] and prostate cancers [225, 226, 227], as well as differentiating the nature of thyroid nodules [228, 229, 230] and lymphadenopathy [231, 232, 233]. Histogram analysis of SE faithfully captures the altered stiffness distribution produced by diffuse fibrosis in chronic pancreatitis. However, the pancreas intrinsically stiffens with age [234]. Consequently, chronological age must be incorporated as a covariate in the interpretation framework to discriminate physiological aging from pathological sclerosis.

SWE, particularly the widely used acoustic radiation force impulse (ARFI) technique, measures tissue elasticity by generating shear waves. The ultrasound probe emits a focused acoustic pulse that induces a localized displacement in the tissue, generating a shear wave. The probe then tracks tissue displacement using echo signals, calculates the shear wave speed, and generates quantitative elastograms overlaid on B‐mode images [235]. However, the accuracy of SWE is affected by the depth of the measurement site due to signal attenuation and dispersion of shear wave velocities, which can vary between devices [236]. Despite these limitations, SWE is widely used for malignancy grading in breast [237, 238] and prostate cancers [239, 240], as well as differentiating the nature of thyroid nodules [241, 242, 243] and lymphadenopathy [244, 245].

Although real‐time elastography, transient elastography and ARFI can currently rule‐out significant interstitial fibrosis after renal transplantation without intervention, none of these UE techniques can yet discriminate individual fibrosis stages with sufficient accuracy. Thus biopsy remains unavoidable, and UE is best reserved for longitudinal surveillance of fibrotic evolution [246]. Conversely, the FSP/STEP model, derived from the combination of UE‐determined liver stiffness and platelet count has demonstrated markedly superior diagnostic performance for S2–S4 hepatic fibrosis compared with conventional serum algorithms (APRI, FIB‐4), offering clinicians a simple, noninvasive and reliable tool for staging liver fibrosis [247].

#### 4.3.2 Magnetic Resonance Elastography

MRE addresses the limitations of subjective manual palpation by expanding the detection range to the entire body. MRE leverages magnetic resonance imaging (MRI) technology, using an actuator to apply continuous low‐frequency shear waves (typically 50–100 Hz) to the target tissue. Shear waves, which are transverse waves with a propagation direction perpendicular to the vibration direction, induce phase shifts in tissue particles that can be detected by motion‐sensitive MRI gradients. These phase shifts are used to generate shear wave propagation maps, from which tissue elasticity parameters are extracted using inversion algorithms and displayed as color‐coded elastograms, providing a visual representation of tissue stiffness [248, 249, 250].

To accommodate different shear wave frequencies, MRE combines various MRI sequences tailored to the hardware limitations of MRI scanners, enhancing signal acquisition efficiency and accuracy while adapting to the elastic properties of different tissues [251, 252]. The most commonly used sequence in clinical practice is the traditional 60 Hz 2D gradient echo sequence [253].

MRE's broad detection range offers unique advantages for assessing the mechanical properties of deep‐seated tumors. It is widely used in the evaluation of liver tumor elasticity [254, 255], preoperative liver regeneration capacity [256], and recurrence risk [257, 258], as well as in the diagnosis of brain [259, 260, 261] and pancreatic tumors [262, 263].

In the aging brain, multifrequency MRE demonstrates a progressive physiological “liquefaction” of otherwise normal parenchyma. Female cortex and white matter retain a significantly higher storage modulus than age‐matched males, implying a biologically younger brain age and establishing MRE as a sentinel modality for subclinical neurodegeneration invisible to conventional imaging [264].

Beyond cerebral aging, both transient elastography and MRE accurately stage advanced fibrosis in primary biliary cholangitis and furnish incremental prognostic value over existing serum assessments [265]. Moreover, focal perihilar stiffening detected by MRE can herald early primary sclerosing cholangitis or congestion before morphological changes emerge [266]. The spatial concordance of these stiffness anomalies with signal alterations on conventional MRI improves the combined detection of both diffuse and focal hepatic disease [266].

#### 4.3.3 Optical Coherence Elastography

OCE is based on optical coherence tomography (OCT) and measures tissue elasticity by introducing mechanical stimulation to induce deformation or displacement within the tissue. OCT utilizes a special low‐coherence light source, such as near‐infrared light, which is split into two beams by an interferometer: a probing beam that enters the imaging tissue and a reference beam. The probing beam, after interacting with the tissue, reflects back and interferes with the reference beam at the detector. By measuring the intensity and phase of the interference signal, OCT reconstructs high‐resolution images of the tissue's internal structure. OCE builds on this foundation by applying mechanical stimuli, such as natural tissue motion or external pressure, to induce minute deformations and thereby obtain local elasticity information [267, 268, 269].

Despite its excellent imaging resolution, OCE is limited by its shallow penetration depth [267]. However, this high resolution makes OCE particularly useful for intraoperative biopsy to assess tumor margins [270, 271, 272], precancerous and cancer diagnosis [273], and the development of site‐specific therapeutic approaches [274].

OCE has likewise emerged as a pivotal modality for fibrosis detection. Quantitative microelastography, employing controlled compression as the loading mechanism, has generated the first micron‐resolution “microstrain maps” of liver [275]. Using an in vitro model of cystic fibrosis in which magnetic nanoparticles are endocytosed by human bronchial epithelial cells, magnetomotive OCE noninvasively and longitudinally quantifies the progressive stiffening of the airway wall [276]. In a mouse model of systemic sclerosis, OCT–OCE demonstrated that dermal stiffening is already significant at 4 weeks, whereas posttreatment skin displayed an intermediate modulus between normal and fibrotic values [277]. Also, an integrated miniature ultrasound–OCT probe coupled with ARF–OCE produced high‐resolution ex vivo elastograms of human carotid plaques and in vitro vessel phantoms, successfully distinguishing the soft, vulnerable core from the stable, fibrous cap and providing a minimally invasive, real‐time mechanical‐imaging tool for early identification of high‐risk atherosclerotic lesions [278].

UE, MRE, and OCE disclose a shared mechanobiological substrate that couples fibrogenesis with oncogenesis. Collagen cross‐linking in fibrosis and stromal stiffening in cancer both elevate Young's modulus, converting tissue hardness into a common imaging biomarker. UE excels in population‐level screening and longitudinal surveillance, exemplified by its ability to guide antiviral timing that aborts the hepatitis–cirrhosis–carcinoma sequence. MRE furnishes three‐dimensional, whole‐organ stiffness maps whose values correlate linearly with pathological collagen deposition, serving as a quantitative reference for both antifibrotic and oncological treatment response. OCE resolves mechanical heterogeneity at micrometer‐to‐millimeter resolution, delivering “biopsy‐grade” elastograms that chart the fibrosis‐to‐cancer transition in vivo. The three are connected across scales to provide synergistic coverage for linked monitoring, risk stratification, and mechano‐targeted intervention across the fibrosis–cancer continuum.

Biomechanical methods have been developed and applied across various scales, from cellular and molecular levels to clinical diagnostics. These methods, based on different devices and principles, offer unique advantages and face specific limitations. Cellular tools provide detailed insights into cellular mechanics and molecular interactions, enabling precise measurements of mechanical properties and forces at the cellular level. Molecular tools further elucidate the mechanobiology of cellular processes by detecting and quantifying molecular‐scale forces and interactions. In the clinical realm, advanced imaging techniques expand the diagnostic capabilities by providing noninvasive assessments of tissue elasticity and mechanical properties. Each method has its own strengths and weaknesses, and the choice of method depends on the specific tissue, organ, and pathological context. Currently, there is ongoing development of higher‐resolution, real‐time wearable, and injectable degradable devices tailored to the unique characteristics and mechanisms of different diseases, aiming to address clinical challenges more effectively.

## 5 Biomarkers in Mechanotransduction as Underlying Therapeutic Targets

Quantifying the mechanical properties of tissues and cells under different diseases situations provide critical insights into how cells interact with microenvironments. In addition, abnormalities in mechanotransduction may disrupt the cell–ECM force balance homeostasis, driving disease progression. Therefore, drugs targeting key molecules in mechanotransduction, also known as mechanical biomarkers, are likely to achieve therapeutic effects by rebuilding the mechanical homeostasis between cells and their microenvironment.

### 5.1 Mechanical Biomarkers in Cancer Progression

In recent years, numerous researchers have begun to decode the mechanism of tumor initiation and progression from mechanobiology perspective, revealing the significant role of mechanical biomarkers in cancer. Mechanical biomarkers mentioned in Table 1 are associated with tumor malignancy, poor prognosis, and resistance to radiotherapy and chemotherapy, highlighting their potential value in drug development. These mechanistic insights are crucial for developing targeted therapies and improving diagnostic strategies, ultimately aiming to enhance patient outcomes in cancer treatment.

**TABLE 1:** Mechanical biomarkers in cancer progression.

Mechanical biomarker	Role in cancer progression	Cancer type	Sensing signal/changing mechanical features	References
Integrin	CAFs align FN by exerting force through integrin α5β1, and prostate cancer cells sense this alignment via integrin αv.	Prostate cancer	Fibronectin aligning	[279]
	LSS promotes Cav‐1‐dependent recycling of integrin β1, enhancing cells migration.	Breast cancer	Low shear stress	[280]
	Integrin β1 autophosphorylation modulates cell invasion.	Breast cancer	Stiffness	[281]
	Integrin β1 contributes to radiotherapy resistance.	Head and neck squamous cell carcinoma	Radiation	[282]
FAK	High collagen density increases matrix stiffness, promoting FAK activation, which in turn activates downstream Rho–ROCK signaling and induces excessive proliferation via ERK, creating a vicious cycle.	Breast cancer	The density of extracellular matrix	[283]
	Following MMP‐9 degradation of fibronectin, the interaction between FAK and Src promotes the invasion of breast cancer cells.	Breast cancer	Degradation of fibronectin	[284]
	Mechanosensing of FAKpY397 originates from the stiffness‐dependent balance of integrin clustering and declustering.	Fibrosarcoma	Stiffness	[285]
	Loss of focal adhesions may facilitate the cytoplasmic‐nuclear translocation of FAK, and the FAK–p53 survival pathway may contribute to tumor progression.	General cancer	Cells detaching	[286]
	The activation of collagen secretion through integrin β1‐FAK in CAFs can maintain cancer stemness.	Pancreatic ductal adenocarcinoma	Type I collagen	[287]
Rho GTPase	The Rho–ROCK signaling pathway and the antagonistic interplay between Rac1 and Rho are closely associated with the dynamics of invasive pseudopods.	General cancer	Matrix plasticity	[288]
	FSS‐induced autophagy is dependent on the PI3K–FAK–Rho GTPases pathway.	Hepatocellular carcinoma	Fluid shear stress	[289]
	Metastatic esophageal cancer cells are shear‐induced activated to form characteristic dynamic membrane blebs via ROCK and Ras signaling.	Esophageal cancer	Fluid shear stress	[290]
	LSS exposure alters the expression of ROCK, p‐MLC, cofilin, and filamin A, rescuing Cav‐1 silencing, which is associated with the formation of lamellipodia.	Breast cancer	Low shear stress	[291]
	CAFs expressing Cav‐1 generate matrix stiffening and alignment, thereby promoting cancer invasion.	General cancer	Matrix stiffening	[292]
Actomyosin	Disrupting the myosin‐dependent contractility of tumor‐initiating cell can resensitize it to soft ECM.	Glioblastoma	Stiffness sensitivity	[293]
	Paclitaxel resistance may be due to overexpression of SEPT9_i1 and its relocalization to microtubules caused by tubulin polyglutamylation, leading to microtubule destabilization.	Breast cancer	Paclitaxel	[294]
	The balance between myosin contractility and oxidative stress‐induced DNA damage/repair is regulated by the Rho–ROCK pathway.	Melanoma	Oxidative stress	[295]
	Entosis is associated with an imbalance of myosin forces between two interacting cells.	General cancer	Cell detaching	[296]
	CTL can locally deform the target tumor surface through actin‐mediated forces at the immunological synapse, enhancing perforin‐mediated cytotoxicity.	General cancer	Physical deformation of the target cell surface	[297]
	In soft tumor repopulating cells, the limited F‐actin content and requirement for perforin to interact with nonmuscle myosin heavy chain 9 prevent the generation of sufficient contractile force to form pores.	General cancer	Stiffness of tumor repopulating cells	[298]
Nucleus	Depletion of emerin causes nuclear shape instability and promotes cancer metastasis.	Prostate cancer	Nuclear shape instability	[299, 300, 301]
	SEPT9_i1 promotes cancer metastasis by stabilizing nuclear Lamin A.	Breast cancer	Nuclear morphology and stiffness	[302]
Transcription‐related factors	Prolonged compression from tumor growth induces nuclear translocation of β‐catenin in adjacent epithelial cells, potentially contributing to tumor progression.	Colon tumor	Prolonged compression	[303]
	Mechanical forces influence cancer progression through the nuclear translocation of YAP/TAZ.	General cancer	Mechanical forces	[304, 305, 306]

#### 5.1.1 Integrins

Integrins play a crucial role in cancer cell migration, invasion, and drug resistance. CAFs can exert forces that significantly modulate the cancer microenvironment and regulate cancer cell migration. CAFs assemble the ECM rich in FN and increase the contractility and traction forces regulated by nonmuscle myosin II and platelet‐derived growth factor receptor α. These forces are transmitted to FN via integrin α5β1, aligning the FN fibers. Prostate cancer cells migrate directionally on CAF‐derived matrices through integrin αv, and this FN alignment is prominent in prostate and pancreatic cancer samples, potentially facilitating cancer cell dissemination [279].

When cancer cells are subjected to shear stress, integrin β1 plays a vital role in adjusting adhesion distribution and movement. Low shear stress (LSS) alters the distribution of integrin β1 in MDA‐MB‐231 cells, activating the ROCK/HDAC6 pathway to downregulate microtubule acetylation. This leads to increased microtubule dynamics, caveolin‐1 (Cav‐1) movement, and Cav‐1‐dependent integrin β1 recycling, thereby promoting focal adhesion turnover and directional cell migration [280].

The phosphorylation of integrin β1 regulates the invasive behavior of breast cancer cells. Through a FRET biosensor, integrin β1 phosphorylation, Shp2 and protein tyrosine phosphatase (PTP)–PEST are detected as negative regulators among 96 PTPs [281]. The NPxY (783/795) sites of integrin β1 are regulated by Src and Arg kinases. Phosphorylated integrin β1 acts as a signal for invasive pseudopod formation, recruiting Dok1 to the cytoplasmic tail and forming a complex with Cofilin, the invasive pseudopod scaffold TKS5, and CTTN. Inhibiting Src or Shp2 significantly suppresses cell migration and invasion [281]. Additionally, integrin β1 phosphorylation increases on softer substrates, contrasting with the overall decrease in tyrosine phosphorylation in softer environments [307], indicating that the role of adhesion signaling in different stiffness contexts warrants further investigation.

Integrins also play a significant role in the organotropism of melanoma metastasis. Integrin α4 and β1 are associated with lymph node metastasis, β3 with lung and bone metastasis, α2 with liver metastasis, and αv with brain metastasis [308]. However, whether different integrin subtypes in the same type of cells represent different organ metastasis tendencies remains unconfirmed. Furthermore, the lack of melanoma therapies targeting integrins may be due to their widespread involvement in normal physiological functions, making it challenging to target only tumor sites. Thus, new strategies targeting universally distributed targets are needed.

Integrins also play a role in cancer radioresistance. Targeting integrin β1 with inhibitory antibodies enhances the radiosensitivity of human head and neck squamous cell carcinoma (SCC) cell lines. This effect is due to FAK dephosphorylation, disassembly of the FAK/cortactin complex, downregulation of the JNK signaling pathway, and induction of cell rounding [282]. Here, integrins are used as a target to alter the mechanical properties of cancer cells, suggesting that identifying more precise mechanical targets in cancer cells could improve their sensitivity to existing therapies such as radiotherapy.

#### 5.1.2 Focal Adhesion Kinase

FAK plays a pivotal role in cancer progression, particularly in the context of the TME. The density of the ECM significantly influences cancer progression. Higher collagen density in the ECM increases matrix stiffness, promoting proliferation and invasion associated with malignant phenotypes in breast epithelial cells [283]. Increased stiffness facilitates the aggregation of activated FAK and the formation of adhesions in 3D matrices, thereby activating downstream Rho–ROCK signaling and ultimately leading to hyperproliferation through ERK [283]. This results in a vicious cycle where increased cellularity further raises the overall density of the microenvironment [283]. FN degradation is also associated with poor prognosis in many cancers. MMP‐9 related to integrin β6 degrades FN, leading to FAK–Src interaction and promoting breast cancer cell invasion and migration through the Erk1/2 and PI3K/Akt/Smad‐1/5/8 pathways [284].

To quantify cellular mechanosensing of substrate stiffness, a model was established to test whether FAKpY397 mechanosensing originates from nanoscale integrin clustering dynamics, stiffness‐dependent integrin clustering and unclustering balance, and FAK Y397 phosphorylation. The relationship between substrate stiffness and FAKpY397 levels is predicted, which is also validated in HT1080 and MDCK cells. This study provides a physical basis for how different cells can specifically sense substrate stiffness through the stiffness–integrin–FAK Y397 phosphorylation cascade [285].

In addition to its kinase activity, FAK can regulate cell behavior through nuclear translocation. During mouse development, FAK promotes Mdm2p53‐dependent ubiquitination and degradation through its FERM domain, independent of kinase activity, thereby maintaining cell survival under stress conditions. When cells detach from the matrix, cytoplasmic FAK levels increase and can translocate to the nucleus. They also discussed several possible mechanisms for FAK nuclear translocation, noting that FAK can form complexes with p53 in both normal and tumor cells [286]. This suggests that focal adhesion loss may promote FAK translocation from the cytoplasm to the nucleus, and the FAK–p53 survival pathway may play a significant role in tumor progression.

The role of nuclear FAK in cancer progression has been further elucidated in SCC, triple‐negative breast cancer (TNBC), and melanoma [309]. In SCC, nuclear FAK interacts with TAF9 in the RNA polymerase II transcription preinitiation complex, promoting the expression of chemokines such as CCL5 [310]. It also facilitates interactions between the transcription factor RUNX1 and the transcriptional repressor Sin3a, thereby downregulating IGFBP3 expression [311]. Additionally, FAK can form complexes with IL‐33, interacting with WDR82 and BRD4 to promote chromatin accessibility [312]. In TNBC, nuclear and cytoplasmic interactions between FAK and NANOG maintain cancer stem cell interactions [313]. In melanoma, FAK nuclear localization and activity are crucial. Myo1E binding to FAK increases FAK activity and promotes its nuclear localization, whereas FAK inhibitors reduce pY397 FAK and nuclear localization [314]. Further research is needed to clarify how kinase‐dependent and kinase‐independent nuclear FAK drives cancer progression in different contexts.

Studies have also shown that small molecule FAK inhibitors or kinase‐inactive FAK mutants significantly promote FAK nuclear localization, which may contribute to tumor resistance or progression [315]. During cancer metastasis, FAK homeostasis is essential for maintaining antiangiogenic [316] and endothelial barrier [317] functions. Additionally, FAK plays a significant role in CAF‐mediated cancer progression. Pancreatic ductal adenocarcinoma cells activate CAFs and increase their type I collagen expression through integrin β1–FAK signaling, maintaining cancer stemness [287]. Pharmacological inhibition or shRNA knockdown of FAK reduces CAF recruitment and fibrosis, thereby suppressing metastasis [318]. Therefore, understanding the differential mechanosensing of FAK in various cells within the TME is crucial for designing targeted therapies.

#### 5.1.3 Rho GTPase

Rho GTPases play a crucial role in cancer cell motility, invasion, and metastatic progression. Therefore, a chemo‐mechanical model is developed that integrates mechanical equilibrium with signaling pathways to explain the formation of invadopodia. The Rho–ROCK pathway and the mutual inhibition between Rac1 and Rho are closely related to invadopodia dynamics, emphasizing the role of matrix plasticity in invadopodia function [288].

Rho GTPases are also essential for cancer cells responding to FSS. Due to the abnormal vasculature and incomplete endothelium in tumor tissues, hepatocellular carcinoma (HCC) cells are directly exposed to blood or lymph flow [319]. In HCC cells, 1.4 dyn/cm^2^ FSS induces autophagy and inhibiting autophagy significantly downregulates PI3K, FAK, and Rho GTPase expression, reducing cell migration [289]. While some highly metastatic tumors may not experience FSS at the primary site, they must enter the circulation and endure FSS during metastasis. Shear stress can induce the formation of characteristic dynamic membrane protrusions in metastatic esophageal cancer cells through ROCK and Ras signaling, causing specific changes in actin and tubulin distribution [290]. Also, LSS exposure promotes cell polarity and focal adhesion dynamics in MDA‐MB‐231 cells, enhancing migration. Cav‐1 silencing significantly reduces stress fiber formation, but LSS exposure can rescue this by altering ROCK, p‐MLC, cofilin, and filamin A expression, while Cav‐1 expression enhances cell motility and LSS triggers rapid lamellipodia formation [291]. These findings suggest that FSS exposure promotes metastasis in these malignancies.

Rho GTPases not only affect cancer cell metastasis but also enable CAFs to remodel the mechanical microenvironment, facilitating tumor invasion and metastasis. The stroma of metastatic breast, renal, colorectal, and melanoma tumors is enriched with Cav‐1‐expressing CAFs. The acellular 3D matrix generated by Cav‐1‐expressing fibroblasts is stiffer and more aligned than that of Cav‐1‐knockout fibroblasts, better stimulating tumor cell elongation, speed, and directional migration. Coculture with cancer cells shows that Cav‐1‐expressing fibroblasts significantly promote tumor cell invasion, which depends on Cav‐1‐regulated p190RhoGAP controlling Rho's downstream force‐dependent behavior [292].

The biophysical phenotype of polyploid giant cancer cells (PGCCs) in late‐stage, high‐grade, or chemotherapy‐resistant tumors has garnered attention. Using multiparticle tracking microrheology to determine the mechanical properties of PGCCs in MDA‐MB‐231, PGCCs exhibit higher inherent cytoplasmic and nuclear stiffness, with significant dysregulation of the RhoA–ROCK pathway and actin cytoskeleton network, and display a slow, persistent migration phenotype [320]. This highlights the potential underestimation of cancer cell biomechanics in advanced, refractory cancers.

Additionally, under oxidative stress, RhoA hydrolysis forms stable amino and carboxyl termini, leading to stress fiber disruption and perinuclear actin rod formation [321]. Since cancer cells often experience oxidative stress, this suggests that abnormal oxidative stress levels may constrain the normal function of cellular mechanical components, potentially triggering aberrant behavior.

#### 5.1.4 Actomyosin

The actomyosin cytoskeleton plays a crucial role in cancer cell mechanosensing of ECM stiffness, migration, and immune cell recognition. Myosin‐generated contractility is a key factor driving tumor metastasis. Pharmacological or genetic activation of RhoA, ROCK, or myosin light chain kinase can restore myosin‐dependent contractility in primary human glioblastoma tumor‐initiating cells, thereby resensitizing them to soft ECM environments [293]. These pathways may serve as potential therapeutic targets or predictive markers for tumor‐initiating capacity within heterogeneous cancer cell populations.

Septins, a highly conserved family of GTP‐binding proteins, play a significant role in modulating Rho GTPases [322]. SEPT9_i1 regulates the cytoskeleton and promotes metastasis in breast cancer [323]. Overexpression of SEPT9_i1 in MCF‐7 cells increased lamellipodia and filopodia formation, enhanced migration and motility, and led to lung metastasis. SEPT9_i1 was found to colocalize with stress fibers, and its overexpression significantly increased stress fiber formation, while knockdown reduced actin fibers and localized them to the cell cortex. Coimmunoprecipitation suggested a close interaction between SEPT9_i1 and paxillin. These findings highlight the possibility of targeting SEPT9_i1 to inhibit metastatic potential in breast cancer.

The cytoskeleton also serves as a sanctuary for aberrantly expressed proteins. Paclitaxel, a common chemotherapeutic agent, stabilizes microtubules by binding to β‐tubulin, inhibiting depolymerization and disrupting mitotic spindle formation, leading to G2/M cell cycle arrest and apoptosis [324]. However, paclitaxel resistance in breast cancer may be due to SEPT9_i1 overexpression and tubulin polyglutamylation, which relocalizes SEPT9_i1 to microtubules and destabilizes them [294]. Thus, eliminating abnormal tubulin modifications and stabilizing microtubules could be crucial for overcoming resistance to existing therapies.

Actomyosin contractility is also influenced by oxidative stress. Melanoma cells possess a fine‐tuned sensor that balances Rho–ROCK‐regulated myosin contractility and oxidative stress‐induced DNA damage/repair. The direction of this balance determines whether cancer cells can spread. When Rho–ROCK‐driven contractility is low, DNA undergoes oxidative stress damage, stabilizing p53 via ATM and inducing specific transcription of DNA damage/oxidative stress response genes, including tumor protein p53‐induced protein 3. This, in turn, activates Rho GTPase activating protein 5, which further inhibits myosin contractility by suppressing ROCK/Rho/MLC2 signaling [295]. This highlights the importance of oxidants in maintaining the cytoskeletal mechanical properties of melanoma cells and suggests that the use of antioxidants in melanoma treatment should be carefully evaluated.

Overholtzer et al. first described entosis in 2007 as a mechanism explaining the common cell‐in‐cell phenotype in human cancers, where one cell invades another, resulting in a live cell being completely engulfed by a neighboring host cell. This process, driven by Rho and ROCK activity, depends on cell–cell junctions and occurs when myosin‐generated forces between two cells are imbalanced. In suspended cells, entosis can be inhibited by matrigel in an integrin β1‐dependent manner, suggesting that entosis may represent an intrinsic tumor suppression mechanism for cells detached from the ECM [296].

Maintaining the mechanical properties of immune cells is crucial for effective immune surveillance. Cytotoxic T lymphocytes (CTLs) can locally deform target tumor surfaces through actin‐mediated forces at the immunological synapse, enhancing perforin‐mediated killing [299]. Variations in cancer cell mechanical properties can lead to immune evasion. CTLs can effectively kill rigid tumor cells but fail to eliminate soft tumor‐repopulating cells (TRCs). Soft TRCs, with less F‐actin, cannot generate sufficient contractility to form perforin pores, but hardening TRCs allows perforin to penetrate the cell membrane and mediate CTL killing [298]. Additionally, high expression of MRTF, which stiffens the actin cytoskeleton, makes melanoma cells more susceptible to CTL attack [325]. The glycocalyx barrier also affects NK cell‐mediated cytotoxicity, with its thickness and compressibility resisting specific cell interactions, possibly related to glycosylation density and sites [326]. Thus, both the mechanical forces of immune cells and the mechanical properties of cancer cells' surfaces or interiors may undergo specific alterations during cancer progression.

#### 5.1.5 Nuclear Scaffold

The nucleus, acting as a mechanosensor that influences cancer cell behavior, is connected to the cytoskeleton via the LINC complex, which plays a significant role in cancer progression. Knockdown of emerin leads to nuclear shape instability and promotes cancer cell metastasis. Evidence of nuclear shape instability was observed in cultured tumor cells, animal models of prostate cancer, human prostate cancer tissues, and CTCs from patients with metastatic disease [299]. Similar findings have been reported in osteosarcoma [300] and breast cancer [301], where emerin deficiency enhances cancer cell invasiveness.

Lamin A/C, which maintains nuclear morphology and stiffness, is also implicated in cancer progression. Invadopodia, structures critical for cancer cell invasion, are preferentially located near the ventral nuclear envelope, suggesting a crucial role for the nucleus in invadopodia function. SEPT9_i1, a key component of invadopodia, influences the initial clustering of invadopodia precursor components such as cortactin and TKS5. Knockout of Lamin A reduced the initial clustering of these components, highlighting the necessity of nuclear stability for invadopodia integrity [302].

Viscoelastic substrates induce changes in nuclear architecture and the epigenome, leading to decreased HDAC activity, increased H3K4‐specific HMT activity, and decreased H3K4‐specific HDM activity [327]. These changes collectively result in elevated levels of euchromatic markers such as AcH3, H3K27ac, and H3K4me3 [327]. The local viscoelastic heterogeneity within tumors is likely to impact the epigenome of cancer cells, influencing their behavior and progression.

#### 5.1.6 Transcription‐Related Factors

The nuclear translocation of transcription‐related factors is a critical mechanism by which mechanical forces influence cancer initiation and progression. The contribution of tumor growth to pressure exerted on adjacent nontumor epithelia are investigated by applying magnetic forces to superparamagnetic liposomes in the mesenchymal cells surrounding colonic crypts in mice, mimicking endogenous early tumor growth pressures (∼1.2 kPa) [303]. Short‐term compression rapidly activated Ret and phosphorylated β‐catenin at Tyr654, weakening E‐cadherin's role in cell adhesion. After 15 days of continuous compression, β‐catenin translocated to the nucleus, and after 30 days, β‐catenin target gene expression increased, leading to aberrant crypt formation. These findings suggest that forces generated by tumor overproliferation may contribute to tumor heterogeneity and promote tumorigenesis.

Multiplex immunofluorescence is used to analyze MRTF‐A/B expression in two breast cancer tissue microarrays finding that most tumor cells in human cancer samples exhibited higher nuclear localization of MRTF‐A/B compared with cytoplasmic localization [328]. Although the ECM is assumed to be the sole activator of MRTF‐A/B, no significant differences in nuclear localization of MRTF‐A/B is found between the invasive front and other regions, unlike p63 and keratin. Thus, the source of MRTF‐A/B nuclear translocation and the role of mechanical forces in this process remain to be elucidated.

YAP/TAZ are key factors in cancer progression, and numerous studies have investigated the mechanoregulation of YAP nuclear translocation. Current cancer therapies targeting YAP/TAZ include inhibiting YAP/TAZ–TEAD interactions, targeting TEAD structure, targeting other YAP/TAZ‐related complex (e.g., BRD4), and targeting upstream mechanical elements (e.g., Rho/ROCK, Src) [304, 305, 306]. However, the development of direct YAP/TAZ inhibitors is limited, likely due to their structure and essential roles in maintaining normal cellular functions. Beyond YAP/TAZ, many other factors exhibit changes in nuclear localization during cancer progression. Although targeting transcription‐related factors is challenging, clarifying their roles in mechanostasis is essential for understanding cancer development.

The alteration of the mechanical microenvironment and the mechanical sensitivity of the affected regions vary significantly during cancer progression, both at the single‐cell level and in tissues or organs. This heterogeneity suggests that the mechanical sensitivity of cancer may differ at various stages of progression and that different types of cancer may have distinct phases of maximal mechanical sensitivity. Moreover, the TME comprises not only cancer cells but also various other cell types, such as CAFs and immune cells, which play crucial roles in cancer progression. This complexity implies that the most mechanically sensitive cell types may vary across different stages of cancer, which is critical for the proper functioning of immune surveillance.

Under mechanical forces, multiple mechanical biomarkers within a cell often change expression, indicating a systemic response rather than an isolated event. Identifying the earliest and most decisive mechanosensitive markers is therefore essential. However, some mechanical biomarkers are indispensable for maintaining basic cellular functions, posing challenges for targeted drug development. Approaches such as targeting upstream regulators or identifying nonessential mechanosensitive markers may offer alternative strategies. In cells, individual components often have multiple roles, and mechanosensitive markers are no exception, also playing important roles in the homeostasis of biochemical pathways. Leveraging biochemical molecules that are minimally involved in mechanosensing within cancer cells to restore the biochemical–mechanical balance and thereby inhibit cancer progression is a promising avenue for future research.

### 5.2 Mechanical Biomarkers in Fibrosis Progression

Fibrosis is the shared pathological hallmark of diverse chronic inflammatory diseases and can affect virtually every organ [329]. It is characterized by the replacement of normal parenchyma with scar‐like connective tissue that is rich in ECM components, leading to progressive architectural disruption and functional impairment [330]. Acute fibrosis may produce scars, keloids or contractures, whereas chronic fibrosis drives irreversible organ stiffening and failure [330]. At present, therapeutic options remain extremely limited, largely because the underlying mechanisms are still incompletely understood [331]. Accumulating evidence indicates that aberrant mechanical phenotype within the cellular microenvironment are a cardinal feature of fibrogenesis. As the attendant mechanotransduction pathways in Table 2 are progressively elucidated, targeting the mechanical biomarkers that sense and generate force is emerging as a novel antifibrotic strategy.

**TABLE 2:** Mechanical biomarkers in fibrosis.

Mechanical biomarker	Role in fibrosis	Organ	References
Integrin	Integrin α6β1 recruits MMP‐2 to degrade type IV collagen, driving fibroblast invasion.	Lung	[332]
	Integrin αvβ6 activates TGF‐β to trigger collagen deposition.	Lung	[333]
	Integrin α3β1 activation drives EMT.	Lung	[334]
	Integrin αvβ1 binds latent TGF‐β1 peptide and activates latent TGF‐β to induce collagen deposition.	Kidney	[335]
	Integrin αv blockade markedly suppresses CCl_4_‐driven fibrosis.	Liver, lung, kidney	[336]
FAK	Mechanical tension activates FAK–ERK to trigger MCP‐1 release, driving hypertrophic scar formation.	Skin	[337]
	FAK expression and activity are markedly upregulated in fibrotic lung lesions.	Lung	[338]
	Mesangial FAK phosphorylation upregulates MCP‐1 via NF‐κB to promote scarring.	Kidney	[339]
Rho GTPase	Stiffness‐activated Rho/ROCK boosts F‐actin polymerization, shuttling MKL1 into the nucleus to sustain myofibroblast phenotype and upregulate BCL‐2, thereby suppressing mitochondrial apoptosis.	Lung	[340]
	RhoA senses liver stiffening from cancer or fibrosis, triggers PI3K–AKT–p300 signaling, and drives HSC‐to‐myofibroblast conversion.	Liver	[341]
	Hepatocyte Rho/ROCK–FAK signaling silences HNF4α network, abolishing hepatocyte‐specific functions.	Liver	[342]
	Rho–p160ROCK drives HSC α‐SMA expression, type I collagen transcription, and collagen deposition.	Liver	[343]
Actomyosin	Actomyosin inhibition prevents fibroblast‐to‐myofibroblast transition.	Lung	[340]
	Thrombin triggers Rho–ROCK to phosphorylate and silence PP1M, elevating actomyosin contraction that widens intercellular gap.	Multiorgan inflammatory state	[344]
	Congestive liver injury imposes cyclic stretch and sinusoidal thrombosis, triggering actomyosin assembly that releases and assembles fibronectin fibers.	Liver	[345]
Nucleus	Dual loss of nesprin‐1 and desmin abolishes nuclear anchoring and tension transfer, igniting TGF‐β/Smad‐driven fibrosis with excess ECM/collagen deposition.	SKELETAL Muscle	[346]
	MAN1 dual‐controls TGF‐β by competing for binding and enzymatically inactivating Smads, thereby setting Smad signal strength and fibro‐osseous susceptibility.	Bone	[347]
	Lamin A/C and emerin jointly tune perinuclear actin polymerization to dictate MKL1 nucleo‐cytoplasmic shuttling.	Heart	[348]
Transcription‐related factors	Mechanical tension on ENFs activates YAP‐dependent mechanotransduction to induce En‐1 expression and convert ENFs into EPFs, driving scar formation.	Skin	[349]
	Integrin β1 signals through PAK and YAP to drive fibrosis.	Liver	[350]
	Nuclear YAP/TAZ, highly expressed in fibrotic lung, independently upregulates PAI‐1 to fuel ECM synthesis, proliferation, and contraction of fibroblasts.	Lung	[351]
	Myofibroblasts sense and amplify tension, shuttling MRTF‐A into the nucleus to cooperate with SRF in upregulating collagen, PAI‐1 and antiapoptotic proteins, thereby establishing a self‐reinforcing fibrogenic feedback loop.	Lung	[352]
	LPA–LPA1 triggers mesothelial cytoskeletal remodeling, shuttling MRTF–A/B into the nucleus to upregulate CTGF, which then paracrinally drives fibroblast proliferation and collagen deposition.	Peritoneum	[353]

#### 5.2.1 Integrins

In pulmonary fibrosis, elevated matrix stiffness is sensed by integrin α6, particularly the α6B splice variant, which cooperates with integrin β1 to recruit MMP‐2 and degrade basement membrane type IV collagen, thereby licensing fibroblast invasion [332]. Integrin αvβ6 is markedly upregulated in human fibrotic lung, localizes to pneumocytes, and serves as the dominant activator of latent TGF‐β. A function‐blocking monoclonal antibody dose‐dependently suppresses collagen deposition in the bleomycin model without exacerbating inflammation, even at subtherapeutic doses [333]. Furthermore, aging can elevate integrin α3β1 expression and downstream tyrosine kinase activity, enhancing formation of the pY654–β‐catenin/pSmad2 complex, lowering the epithelial–mesenchymal transition (EMT) threshold and sustaining aberrant alveolar epithelial cell EMT, which is a key driver of scar perpetuation in idiopathic pulmonary fibrosis [334]. Meanwhile, the ECM component laminin‐111, traditionally viewed as a basement membrane scaffold, is dynamically processed by MMP‐2 to generate a β1‐chain fragment that signals via integrin α3β1–EMMPRIN to downregulate MMP‐2, upregulate MMP9 and E‐cadherin, and thereby trigger EMT in embryonic stem cells, illustrating a conserved mechanochemical loop exploitable by fibrotic lesions [354].

In renal fibrosis, activated PDGFR β^+^ fibroblasts are critically dependent on integrin αv family members. Integrin αvβ1 binds the latency‐associated peptide of TGF‐β1 and catalyzes its activation. Small molecule inhibition of this interaction reduces collagen deposition in the unilateral ureteral obstruction model and reduces BUN levels in adenine‐induced chronic kidney disease, positioning integrin αvβ1 as a therapeutic node in renal fibrosis [335].

Pdgfrb–Cre‐mediated deletion of the integrin αv subunit in hepatic stellate cells (HSCs) abrogates assembly of αv‐containing heterodimers and concurrently suppresses CCl_4_‐driven liver, lung, and kidney fibrosis. The small‐molecule inhibitor of αv named CWHM 12 reverses established fibrosis when administered after injury [336].

Collectively, integrins function as master switches in the mechanochemical dialogue of fibrosis: clustering of some subtypes can activate TGF‐β and reinforce β‐catenin/Smad signaling to create a self‐amplifying cycle of collagen deposition and EMT. Genetic or pharmacological blockade of the subfamily simultaneously halts scar progression in mature models of lung, liver and kidney fibrosis, validating the potential feasibility of a single‐target, multiorgan therapeutic strategy.

#### 5.2.2 Focal Adhesion Kinase

Mechanical tension activates FAK via integrin clustering. Then, downstream ERK signaling drives fibroblast secretion of monocyte chemoattractant protein‐1 (MCP‐1), recruiting inflammatory cells and amplifying scar hypertrophy. Genetic knockout or pharmacological inhibition of FAK markedly attenuates hypertrophic scarring in mice, identifying FAK as the critical mechanosensitive switch that couples physical force to fibrotic output [337].

In human pulmonary fibrosis, FAK expression and phosphorylation are markedly elevated within fibroblastic focal. Pharmacological blockade or siRNA‐mediated silencing of FAK prevents bleomycin‐induced lung fibrosis in vivo and abrogates endothelin‐1‐stimulated fibroblast adhesion, contractility and profibrotic gene expression, demonstrating that FAK serves as the central hub linking matrix adhesion to the fibrotic phenotype [338].

Glomerulosclerosis, the hallmark of progressive glomerulonephritis, is characterized by aberrant mesangial ECM accumulation. In mesangial cells, ECM–integrin engagement triggers FAK phosphorylation and subsequent NF‐κB‐dependent upregulation of MCP‐1, thereby perpetuating inflammatory cell recruitment and scar formation. This FAK‐mediated axis provides an early cellular mechanism explaining the self‐amplifying cycle of matrix deposition, inflammation and fibrosis that underlies glomerulosclerosis [339].

Across lung, kidney and other injured organs, integrin sensing of matrix stiffening promotes focal clustering, followed by FAK‐driven signaling that induces myofibroblast differentiation and MCP‐1 release, establishing a positive feedback loop among matrix rigidity, inflammation, and scarring.

#### 5.2.3 Rho GTPase

Across chronic fibrotic organs, progressive stiffening and myofibroblast apoptosis‐resistance are locked in a self‐reinforcing loop anchored by Rho/ROCK. Tension‐induced F‐actin polymerization enhances nuclear translocation of the mechanosensitive coactivator MKL1, which sustains α‐SMA expression and upregulates BCL‐2 to silence mitochondrial apoptosis. Genetic ablation of MKL1 or fasudil breaks this feedback, triggering myofibroblast apoptosis and markedly attenuating lung fibrosis [340].

Liver fibrosis and oncogenesis are mechanistically intertwined. When RhoA senses pathological stiffening, it ignites a PI3K–AKT–p300 cascade that converts quiescent HSCs into collagen secreting myofibroblasts, simultaneously generating tumor‐promoting desmoplasia [341]. Within HSCs, matrix rigidity drives RhoA–ROCK‐dependent phosphorylation of p300, amplifying profibrotic transcription and ECM deposition [341]. In hepatocytes, the same Rho/ROCK axis suppresses the HNF4α network via FAK‐mediated signals, extinguishing liver‐specific functions [342]. Dual pharmacologic blockade of Rho/ROCK therefore ablates myofibroblast activation while restoring hepatocyte identity, offering a rational treatment strategy for fibrosis‐cirrhosis. In dimethylnitrosamine‐induced liver fibrosis, Rho–p160ROCK signaling is indispensable for HSC activation. Rho–GTP promotes α‐SMA transcription, type I collagen synthesis, and hydroxyproline accumulation [343]. Oral administration of Y‐27632 abolishes these responses in vivo, confirming ROCK as a druggable node [343]. Nevertheless, the systemic safety profile of first generation ROCK inhibitors remains equivocal. Next generation compounds with enhanced isoform selectivity or tissue selectivity are urgently required to translate mechano‐targeted Rho/ROCK therapy into clinical antifibrotic practice.

#### 5.2.4 Actomyosin

In idiopathic pulmonary fibrosis, the heightened sensitivity of myofibroblasts to ROCK inhibitors is coupled to a reduction in F‐actin content and a shutdown of the actomyosin contractile apparatus. And then MKL1 is unable to translocate from cytosol to nucleus, leading to downregulation of the antiapoptotic protein BCL‐2, activation of the intrinsic apoptotic pathway and a blockade of normal fibroblast‐to‐myofibroblast differentiation [340].

Thrombin‐induced endothelial‐barrier breakdown is mediated by the Rho–ROCK axis, which phosphorylates and inhibits the PP1M phosphatase, thereby increasing myosin light chain phosphorylation and driving actomyosin contraction that transiently widens intercellular gaps. A ROCK‐independent pathway simultaneously generates larger paracellular pores responsible for sustained vascular hyperpermeability. Administration of the ROCK inhibitor Y‐27632 prevents stress fiber assembly and partially restores barrier integrity, indicating that pore contraction is ROCK dependent, whereas the mechanism underlying paracellular pore formation remains to be elucidated [344].

In congestive liver injury, cyclic mechanical stretch imposed by sinusoidal dilatation synergizes with fibrin‐rich thrombi to activate HSCs. Both stimuli are sensed by integrin β1, triggering the assembly and activation of actomyosin contractile units that promote FN release and fibrillar assembly, thereby amplifying the fibrogenic signal. Blocking integrin β1 or depolymerizing F‐actin abolishes this response, establishing actomyosin‐mediated mechanotransduction as a critical hub in congestion‐driven hepatic fibrosis [345].

These data consolidate the integrin–Rho/ROCK–actomyosin axis as a unifying mechanosignaling chain within the fibrotic microenvironment. Mechanical force through ECM or stretching is first detected by membrane integrins, which recruit Rho GTPases and their downstream effector ROCK. Activated ROCK phosphorylates myosin light chain, promoting F‐actin polymerization and stress fiber assembly, thereby enhancing actomyosin contractility and triggering downstream changes in cellular mechanics and matrix secretion.

#### 5.2.5 Nuclear Scaffold

In skeletal muscle, the LINC complex component nesprin‐1 and the intermediate filament protein desmin jointly secure nuclear positioning and mechanochemical coupling. Concomitant knockout of nesprin‐1 and desmin collapses nuclear anchoring forces and disrupts tensile transmission, unleashing a fibrogenic cascade characterized by excessive collagen/ECM deposition, elevated passive stiffness and contractile failure [346]. Thus, nuclear tethering proteins not only dictate nuclear placement but also act as gatekeepers that restrain muscle fibrosis.

MAN1, encoded by LEMD3, is an inner NM protein that competes with FAST1 for Smad2/3 binding, occluding their nuclear import, and simultaneously recruits the phosphatase PPM1A to catalyze Smad2/3 dephosphorylation, thereby preventing Smad4 complex formation. Haplo‐insufficiency or loss‐of‐function mutations in LEMD3 reduce MAN1 dosage, uncage TGF‐β/Smad signaling, and precipitate progressive osseous sclerosis plus multisystem developmental anomalies [347]. MAN1 therefore operates through competitive binding and enzymatic inactivation to negative rheostat of TGF‐β signaling, with its expression level dictating Smad output and the susceptibility to fibro‐osseous disease.

During LMNA‐linked skeletal and cardiac myopathies, lamin A/C cooperates with the inner‐nuclear‐membrane protein emerin to govern perinuclear actin polymer dynamics that gate the nucleo‐cytoplasmic shuttling of the mechanosensitive transcription regulator MKL1 [348, 355]. LMNA deletion or the pathogenic N195K mutation disrupts this complex, provokes maladaptive remodeling of the intranuclear F‐actin network, blocks MKL1 nuclear import and blunts downstream cardiopoietic gene expression, culminating in developmental and functional cardiac deficits [348]. Exogenous emerin re‐establishes perinuclear actin homeostasis and restores MKL1 nuclear localization, validating the lamin–actin–MKL1 axis as a mechanotransduction checkpoint whose correction can rescue LMNA‐associated cardiomyopathic phenotypes.

#### 5.2.6 Transcription‐Related Factors

Adult cutaneous wound healing culminates in either fibrotic scarring or scar‐less regeneration, a fate dictated by the Engrailed‐1 (En‐1). Mechanical tension within the wound activates a YAP‐dependent mechanotransduction cascade that converts En‐1‐negative fibroblasts (ENFs) into En‐1‐positive profibrotic fibroblasts (EPFs), thereby driving scar formation. Conversely, pharmacological or genetic inhibition of YAP precludes En‐1 activation, preserves the ENF regenerative phenotype, and restores skin appendages, ultrastructure, and biomechanical strength [349]. The delineation of the YAP–En‐1 axis thus establishes a direct molecular link between mechanical input, transcriptional reprogramming and fibrosis, providing a rational basis for clinical scar‐less healing via YAP/En‐1 targeting.

In hepatic fibrosis, the scar ECM is generated primarily by myofibroblasts that sense pathological stiffness through integrin β1, which frequently paired with integrin α11. This mechano‐chemical circuit is underpinned by PAK and the mechanosensitive cotranscriptional regulator YAP1. YAP1 reciprocally sustains integrin β1 expression while driving ECM secretion, proliferation, migration, and contractility. Pharmacological inhibition of either PAK or YAP1 markedly ameliorates experimental liver fibrosis, with PAK inhibitors producing the most rapid suppression of the myofibroblast phenotype and limiting postinjury scar expansion [350]. Thus, the integrin β1–PAK–YAP1 module constitutes a therapeutically tractable axis for reversing hepatic fibrosis.

Pathological matrix stiffness promotes nuclear accumulation of YAP/TAZ, which are markedly elevated in fibrotic human lung tissue. YAP/TAZ directly upregulate plasminogen activator inhibitor‐1 (PAI‐1) in a TGF‐β‐independent manner, fueling fibroblast ECM synthesis, proliferation, and contraction. Gene silencing or pharmacological blockade of YAP/TAZ selectively abrogates these profibrotic functions under stiff conditions, whereas transgenic activation of YAP/TAZ is sufficient to trigger ECM deposition even in soft matrices or intact mouse lung [351]. Consequently, YAP/TAZ function as master transcriptional related nodes that perpetuate a stiffness–YAP/TAZ–ECM feed‐forward loop, offering a theoretical framework for disrupting fibrosis self‐amplification through YAP/TAZ inhibition.

During pulmonary fibrogenesis, myofibroblasts amplify mechanical tension via stress fibers. The core mechanism involves nuclear translocation of the transcriptional coactivator MRTF‐A, which partners with SRF to upregulate collagen, PAI‐1, and antiapoptotic proteins, thereby establishing a self‐reinforcing profibrotic circuit [352]. The small‐molecule inhibitor CCG‐203971, which selectively blocks MRTF‐A nuclear import, reverses TGF‐β1‐induced myofibroblast differentiation and enhances apoptosis sensitivity in vitro [352]. In both bleomycin injury and alveolar type II cell injury mouse models, CCG‐203971 concurrently reduces lung collagen and PAI‐1 levels while inducing myofibroblast apoptosis, significantly attenuating fibrotic burden [352]. Collectively, the MRTF‐A/SRF axis integrates mechanical signaling with transcriptional output and represents a critical molecular switch driving and sustaining pulmonary fibrosis.

In experimental peritoneal fibrosis, lysophosphatidic acid (LPA) engages its receptor LPA1 to trigger cytoskeletal remodeling in mesothelial cells, promoting nuclear translocation of MRTF‐A/B and upregulation of connective tissue growth factor (CTGF). CTGF then acts in a paracrine fashion to stimulate fibroblast proliferation and collagen deposition. Genetic or pharmacological blockade of LPA1 disrupts this LPA–LPA1–MRTF–CTGF chain, reducing collagen abundance [353]. Thus, the signaling axis directly couples lipid signaling to fibrogenesis, providing both conceptual and experimental justification for combined antifibrotic strategies that target LPA1 or MRTF.

Increased stiffness of fibrotic tissue ignites and sustains a mechanotransduction‐centered positive‐feedback loop. Integrin clustering senses ECM rigidity and triggers FAK–Src and Rho–ROCK signaling, heightening actomyosin tension. The generated force is transmitted through the cytoskeleton–LINC complex to the nuclear envelope, promoting nuclear import of YAP/TAZ and MRTF‐A. These transcriptional coactivators upregulate collagen, CTGF, PAI‐1, and other profibrotic genes, thereby self‐amplifying fibrosis while supplying proproliferative cues to neighboring cells. Consequently, fibrosis can function as the mechanical scaffold of the TME. In aging tissue, cumulative ECM cross‐linking and chronic tensional imbalance perpetuate the same pathway, potentially driving the SASP. Interruption of this mechanotransduction axis therefore offers a unified strategy to suppress fibrosis, lower cancer risk and delay tissue aging.

### 5.3 Mechanical Biomarkers in Aging Progression

Systemic aging is occasionally accompanied by fibrotic remodeling, yet a strict cause‐and‐effect link is lacking. Whether organ‐level stiffness changes detected by clinical elastography truly mirror organismal biological age therefore remains equivocal. In contrast, single‐cell mechanophenotyping in Table 3 directly captures the incremental mechanical alterations that occur within senescent cells. So its integration with molecular biology toolkits may permit dissection of the causal pathways that underlie these age‐related mechanical shifts.

**TABLE 3:** Mechanical biomarkers in aging.

Mechanical biomarker	Role in aging	Cell type	References
Integrin	Loss of functional integrin α5β1 heterodimers desensitizes senescent cells to fibronectin‐induced DNA synthesis and proliferation.	Fibroblast	[356]
	Aged fibroblasts retain integrin α2 levels comparable to young cells, yet α2β1‐dependent migration is severely impaired and coincides with disorganized actin cytoskeleton.	Fibroblast	[357]
	Young cartilage expresses only integrin‐β1α3 and β1α5, whereas aging or dedifferentiation deposits type I collagen thick fibrils and induces de novo β1α1 and β1α2 receptors on the cell surface.	Chondrocyte	[358]
	Aging upregulates integrin β3, which drives fibroblasts into a development‐like senescent phenotype.	Fibroblast	[359]
	Blocking integrin signaling rescues age‐waning βPIX–GIT and prevents p16^Ink4a^–pRb‐driven senescence.	Fibroblast	[360]
FAK	Caveolin‐1 overexpression couples integrin–FAK signaling to Rac1/Cdc42‐driven actin remodeling, switching fibroblasts from young spindle to senescent flat spreading morphology.	Fibroblast	[361]
	Mechanical stretch amplifies integrin–FAK–Src–Ras signaling to activate p38 MAPK and drive neonatal rat cardiomyocyte hypertrophy.	Cardiomyocyte	[362]
	Aging aortae upregulate FAK and adhesion‐complex proteins, yet high‐pressure fails to induce FAK phosphorylation in very aged group,	Vascular smooth muscle cell	[363]
	Senescent cells have lower c‐Src, which in growing cells activates its tyrosine kinase and binds phosphorylated FAK and paxillin.	Fibroblast	[364]
	In aged skeletal‐muscle feed arteries, pFAK397 in VSM are markedly downregulated, leaving cells unable to transmit contractile tension effectively.	Vascular smooth muscle cell	[365]
Rho GTPase	Aged mouse CD4^+^ T cells show elevated Rac but sharply reduced RhoA activity, likely crippling immune synapse formation and function.	CD4^+^ T cell	[366]
	Angiotensin II activates BMEC and HSC/P Rho GTPase–actin signaling, dampens integrin activity, and releases stem cells into the periphery, causing premature.	Hematopoietic stem cells	[367]
	hESC dissociation creates an Abr‐driven Rho‐high/Rac‐low imbalance that hyperactivates ROCK–actomyosin and triggers anoikis.	Embryonic stem cells	[368]
Actomyosin	Actomyosin inhibition rescues OPC proliferation and differentiation on stiff, aging‐mimicking substrates.	Oligodendrocyte precursor cell	[369]
	Aging shifts the G‐/F‐actin balance toward increased F‐actin.	Fibroblast	[150]
	Lymphocytes from healthy elderly subjects display elevated basal actin levels but stimulus‐induced actin polymerization is lower.	Lymphocytes	[370]
	Aging reduces T‐cell membrane fluidity while raising F‐actin levels, curtailing lamellipodia formation and impairing immune synapse assembly and activity.	CD4^+^ T cell	[371]
	In aged mouse alveolar macrophages, attenuated F‐actin polymerization impairs filopodia formation and reduces cell‐surface expression of the bacterial scavenger receptor MARCO.	Macrophage	[372]
Nucleus	In progeroid mice, Sun2 overexpression tightly couples cytoskeleton to nucleoskeleton, provoking nuclear damage.	Mesenchymal stromal cell	[373]
	Lamin A/C expression decreases and disperses around the nucleus in aged cells.	Cardiomyocyte	[374]
	Lamin A/C is markedly reduced in aged cells.	Osteoblast	[375]
	In senescent dermal fibroblasts, cryptic activation of the LMNA exon 11 splice site generates aberrantly lamin A.	Fibroblast	[376]
	In HGPS, lamin A mutation downregulates EZH2, erases H3K27me3/H3K9me3, dismantles facultative and centromeric heterochromatin, and derepresses satellite III transcription.	Fibroblast	[377]
	Histone demethylase inhibition rescues the HGPS phenotype.	Fibroblast	[378]
	Aging downregulates endothelial Nup93, disrupts nuclear pore integrity, traps YAP in the nucleus, and fuels inflammation and senescence.	Endothelial cell	[379]
Transcription‐related factors	In aged progenitors YAP/TAZ switch on only at super‐physiological stiffness, shifting activity from myoepithelial to luminal cells.	Luminal progenitor cells	[380]
	Aging enlarges endothelial cells, due to the increasing of CDC42 and trapping YAP1 in cytoplasm to block proliferation and blunt angiogenesis.	Endothelial cell	[381]
	Aged muscle's straighter, stiffer collagen activates fibroblast YAP/TAZ, which secrete profibrotic matrix and switch MuSCs from myogenesis to fibrogenesis.	Muscle stem cell	[382]
	Aging skeletal muscle downregulates MRTF‐A.	Skeletal muscle cell	[383]
	Aging blunts YAP/TAZ mechanotransduction, downregulating lamin B1 and ACTR2 transcription, compromising nuclear envelope integrity, and activating the cGAS–STING pathway to drive tissue senescence and inflammation.	Fibroblast	[384]

#### 5.3.1 Integrins

In both late‐passage normal and Werner syndrome progeroid fibroblasts, FN mRNA is upregulated, yet its principal receptor, integrin α5β1, undergoes reduced quantity and functional attrition. α5‐Polypeptides level falls and a fraction of the subunit is sequestered, lowering the density of functional α5β1 heterodimers at the membrane. Although total β1 remains constant, its intracellular processing slows. Consequently, aged cells adhere poorly to FN and lose the ability to mount FN‐stimulated DNA synthesis and proliferation [356].

Comparing migratory capacity, MMP secretion, substrate anchorage, and integrin α2β1 function of fibroblasts from four young versus six old healthy donors revealed that α2 abundance is preserved, but migration‐defective senescent cell lines display a marked loss of α2β1 activity that parallels actin cytoskeletal disarray rather than altered MMP/TIMP profiles, implicating cytoskeletal disorganization in cellular aging [357].

Aging chondrocytes exhibit an integrin shift‐work pattern. Young cartilage expresses only α3β1 and α5β1, whereas senescence/dedifferentiation and the attendant deposition of type I collagen thick fibrils induce de novo appearance of α1β1 and α2β1 receptors, achieving a new collagen matched to new‐integrin model [360].

Integrin β3 rises with age in a ligand‐independent manner and triggers the processing similar to developmental senescence. CBX7/PRC1‐mediated epigenetic erosion derepresses ITGB3. Conversely, silencing integrin β3 reverses senescence hallmarks and suppresses the inflammatory SASP in human aged fibroblasts, identifying integrin β3 as a candidate target for tissue homeostasis and antiaging intervention [361].

Aging‐dependent decline of βPIX–GIT relieves competitive inhibition of paxillin–calpain, leading to AMPH1 cleavage, clathrin‐mediated integrin endocytosis blockade and sustained Rac–ROS signaling that activates p16^Ink4a^–pRb senescence. Interrupting integrin signaling reverses this phenotype in vitro and in vivo, offering an additional antiaging node [360].

Given the broad distribution of integrins and their upstream position in many biochemical cascades, which albeit with tissue‐restricted patterns, their sensitivity to matrix stiffness heterogeneity remains undefined. Therefore, when evaluating integrins as drug targets, matrix mechanics should be incorporated as a filtering parameter.

#### 5.3.2 Focal Adhesion Kinase

Cav‐1 overexpression couples integrin–FAK signaling to Rac1/Cdc42‐driven actin remodeling, synchronously reinforcing focal adhesions and stress fibers and converting young, spindle‐shaped fibroblasts into senescent, flattened cells that form hyperstable ECM contacts, which is an indirect index of aging‐associated fibrotic deposition [361].

Enhanced mechanical stretch activates the integrin–FAK–Src–Ras–p38 MAPK cascade to promote neonatal cardiomyocyte hypertrophy. Because the same axis is reactivated during aging, chronic p38 MAPK may be stimulated in senescent myocardium [362].

In aging aorta, FAK and adhesion complex proteins are upregulated, yet high pressure fails to induce FAK (Tyr925) phosphorylation in very aged group, indicating an aging‐dependent decline in FAK mechanotransduction capacity that must be assessed by both abundance and activity [363].

Systematic comparison of young versus senescent dermal fibroblasts reveals reduced c‐Src expression with aging. Since active c‐Src phosphorylates FAK and paxillin in proliferating cells, maintenance of FAK activity may oppose cellular aging [364].

In skeletal‐muscle feed arteries of aged rodents, pFAK‐397 in VSM are markedly downregulated, accompanied by smaller focal adhesions and diminished SMα‐actin stress fibers. Although integrin α5β1‐mediated matrix adhesion increases, defective FAK signaling prevents effective tension transmission, blunting vasoconstrictor responses to phenylephrine and Ang II and representing a key mechanism of age‐related vascular dysfunction [365].

Current data on FAK activity and organismal aging may be contradictory. On one hand, FAK can accelerate senescence by reinforcing cytoskeletal tension, promoting proinflammatory cytokine release and tissue fibrosis. On the other hand, FAK sustains adhesion homeostasis, and these effects are compatible with delayed senescence and preserved tissue integrity. These results reflect the pleiotropy of cell types, aging stages, and signaling environments, underscoring the need for spatiotemporally resolved network analyses to clarify the specific role of FAK in aging.

#### 5.3.3 Rho GTPase

In aged mouse CD4^+^ T cells, total RhoA and Rac levels remain unchanged, yet Rac activity rises while RhoA activity declines. This shift parallels aging‐related dephosphorylation of the ezrin–radixin–moesin and correlates with defective immune synapse formation and diminished T cell responsiveness, suggesting that waning immune‐cell mechanics may contribute to age‐associated oncogenesis [366].

High angiotensin II activates the Rho GTPase–actin axis in bone‐marrow endothelial cells (BMEC) and hematopoietic stem/progenitor cells (HSC/P), attenuating integrin function and precipitating stem‐cell detachment and peripheral mobilization. Inhibition of RhoA or blockade of Ang‐II signaling reverses this process, revealing reciprocal Rho–integrin crosstalk and positioning Rho GTPases as master switches for vascular disease‐linked, premature HSC release [367].

Dissociation of human embryonic stem cells (hESCs) elicits an Abr‐mediated “high‐Rho/low‐Rac” imbalance that hyperactivates ROCK–actomyosin and triggers anoikis. Activation of Rho/ROCK with suppression of exogenous Rac restores adhesion and viability causing by Abr‐depleted, demonstrating that Rho‐dependent actomyosin tuning governs hESC anchorage and operates as a critical switch between survival and dissociation‐induced, aging‐like apoptosis [368].

#### 5.3.4 Actomyosin

With aging, the prefrontal cortex gradually stiffens. When oligodendrocyte precursor cells (OPCs) are plated on soft polyacrylamide hydrogels that mimic young brain elasticity, their proliferative capacity is restored. Conversely, OPCs cultured on stiff substrates recover proliferation only after actomyosin inhibition, and this rescue requires the mechanosensitive Ca^2+^ channel PIEZO1, implicating actomyosin‐mediated stiffening in the aged OPC phenotype [369].

Flow‐cytometric quantification of G‐actin and F‐actin (Alexa‐647‐phalloidin vs. Alexa‐488‐DNase I) in fibroblasts from young and old donors reveals a shift toward F‐actin in senescent cells, indicating that age‐dependent cytoskeletal contractility is coupled to altered actin partitioning [150].

Lymphocytes from healthy elderly subjects display elevated basal actin levels but stimulus‐induced actin polymerization is lower, suggesting that impaired actin dynamics may be a shared mechanism underlying multilineage functional decline during aging [370]. Aging decreases T cell membrane fluidity while increasing F‐actin content, curtailing lamellipodia formation and compromising immune‐synapse assembly and signaling [371]. Aged mouse alveolar macrophages exhibit diminished F‐actin polymerization, fewer filopodia and reduced surface expression of the scavenger receptor MARCO, correlating with impaired bacterial clearance and linking actin dysdynamics to increased infection susceptibility in the elderly [372].

Cells that rely on dynamic pseudopodia for migration or phagocytosis undergo age‐related remodeling of pseudopod architecture, turnover rate and biosynthetic cost, leading to system‐wide stiffness changes that define the mechanophenotype of senescence. How myosin II remodeling contributes to this process remains to be elucidated.

#### 5.3.5 Nuclear Scaffold

In Zmpste24‐KO progeroid mice, Sun2 is markedly upregulated in gastrocnemius mesenchymal stromal cells, tightening the cytoskeletal‐nuclear scaffold and provoking blebbing, DNA damage, heterochromatin loss, and telomere detachment. Sun2 knock‐down relieves extreme nuclear strain and restores progeroid cell function [373].

Aged mouse cardiomyocytes exhibit dispersed, low‐level lamin A/C at the nuclear rim, but a causal link to heart failure remains unresolved [374]. Similarly, osteoblasts from aged mice show reduced lamin A/C, whereas young osteoblasts retain high lamin B expression [375].

In senescent dermal fibroblasts, cryptic splicing within LMNA exon‐11 generates a Δ50 lamin A that aggregates aberrantly at the nuclear periphery while canonical lamin A disappears [376]. In Hutchinson‐Gilford progeria syndrome (HGPS), mutant lamin A downregulates EZH2, erases H3K27me3/H3K9me3, collapses facultative heterochromatin and derepresses satellite III transcription, driving premature aging [377]. Histone demethylase inhibition restores heterochromatin and normalizes nuclear morphology independently of lamins, rescuing the HGPS phenotype [378].

Aging also reduces endothelial Nup93, disrupting nuclear pore complex integrity, promoting YAP nuclear accumulation and vascular inflammation. Nup93 restoration or YAP inhibition reverses this senescent vasculopathy [379].

Collectively, aging‐dependent alterations in nuclear envelope and nuclear pore proteins compromise nuclear mechanics, manifesting as blebbing, chromatin remodeling, epigenetic rewiring, telomere mis‐localization, DNA damage, and impaired transcription factors trafficking, which can feedback to accelerate aging.

#### 5.3.6 Transcription‐Related Factors

Aging blunts the mechanosensitivity of human mammary epithelial progenitors. Whereas young cells deploy YAP/TAZ to sense physiological stiffening and commit to the myoepithelial lineage, aged progenitors activate YAP/TAZ only at supra‐physiological rigidities and redirect the response from myoepithelial to luminal fates [380].

The rise in Cdc42 and the decrease in nuclear location of YAP1 cause aging endothelia to enlarge, suppressing proliferation and underlying angiogenic decline. Normalizing cell size, inhibiting Cdc42, or forcing YAP1 nuclear entry reinstates vessel sprouting, identifying a cell size–Cdc42–YAP1 axis that governs senescent neovascularization [381].

Aged muscle displays increased ECM stiffness and decreased collagen tortuosity, activating fibroblastic YAP/TAZ and driving secretion of profibrotic matrix. Muscle stem cells (MuSCs) forfeit myogenic potential and adopt a fibrogenic fate [382]. In senescent skeletal muscle, nuclear MRTF‐A expression decline, indicating that waning SRF–MRTF signaling is a molecular driver of geriatric muscle atrophy [383].

Progressive aging dampens YAP/TAZ mechanotransduction, eroding transcriptional maintenance of lamin B1 and ACTR2, compromising nuclear envelope integrity, unleashing cGAS–STING signaling and fueling tissue senescence and inflammation. Sustained YAP/TAZ activation or STING inhibition arrests this cascade and delays aging phenotypes [384].

During aging, although the external stiffness changes, cells exhibit a reduced sensitivity to it. Elevated tension initially promotes nuclear translocation of YAP/TAZ in young cells, driving proliferation and inflammatory programs. However, persistent high tension or adhesion instability triggers DNA‐damage responses that enforce cellular senescence. Concurrently, aging shifts integrin expression profiles, alters FAK phosphorylation dynamics, and unbalances RhoA–ROCK activity, leading to aberrant actomyosin contractility. These changes deform nuclear lamin A/C, remodel chromatin, and restrict nuclear pores, further impairing mechano‐to‐transcription coupling and establishing a negative force‐gene feedback loop that underlies the increased stiffness, impaired migration, and diminished phagocytic capacity characteristic of aged cells.

In oncology, mechanical biomarkers have been intimately linked to extracellular mechanical cues and intensively interrogated, yet most studies remain confined to the cellular scale. Nevertheless, biomechanical analyses have already illuminated key shortcomings of current therapeutic strategies. In fibrosis, mechanical biomarkers primarily serve as indicators of disease progression. However, the safety margins and improvements of mechanically targeted drugs still require ongoing attention. Mechanobiological biomarkers associated with aging are also primarily detected at the single‐cell level, but organismal aging is not only manifested in the remodeling of the cytoskeleton but also in hierarchical changes at the tissue and organ levels. At meso‐ and macro‐scales, cellular aging states can exhibit pronounced heterogeneity. Cancer, fibrosis, and aging are further intertwined: whether the fibrogenic programs activated during tumorigenesis and organismal aging share biomechanical driving factors, and whether distinct mechanotransduction features generate different senescent microenvironments in the tumor core versus the adjacent stroma, remain unresolved and highly pertinent questions.

### 5.4 Underlying Therapeutic Targets and Related Drugs

Regarding the key molecules in the mechanotransduction pathways mentioned before, some existing drugs have already entered clinical trials, but many key targets still face numerous challenges in drug design. Even when corresponding inhibitors exist, they can only be used as preclinical tools. Meanwhile, some molecules play important roles in other signaling pathways. Although there are already approved clinical drugs targeting them, their use in cancer and other diseases still requires evaluation through clinical trials.

A variety of drugs can be developed targeting mechanotransduction, including small molecule compounds, peptides, antibodies, antibody drug conjugates and peptide drug conjugates, nanomedicines, CAR‐T cell therapies, and imaging agents. Currently, there are relevant reviews summarizing the role of integrins in various diseases and the prospects and challenges of developing integrin‐targeted drugs [385, 386]. Some recent clinical trials targeting integrins in solid tumors are concluded in Table 4. As for FAK, several clinical drugs have already been marketed, and developing targeted FAK inhibitors is a promising therapeutic strategy [309, 387, 388]. However, drugs targeting Rho GTPases themselves are still at the preclinical inhibitor stage [389, 390, 391]. Although targeting Rho GTPases, RhoGEFs, and RhoGAPs remains challenging [392], there are currently approved drugs targeting the downstream ROCK, but their research related to cancer is still at the clinical trial stage. Additionally, research on drugs targeting the downstream MRTF is also limited to preclinical inhibitors [393, 394, 395, 396, 397]. YAP/TAZ, although extensively studied in the Hippo pathway and already recognized for their mechanosensitive roles, still have drugs in preclinical or clinical development stages [398]. Drugs affecting actin polymerization are still limited to the preclinical small molecule stage [399, 400, 401]. Traditional small molecule inhibitors targeting nonmuscle myosin II are highly toxic [402], but improved versions have recently entered clinical trials.

**TABLE 4:** Therapeutic targets in cancer. *Note*: Unreferenced trials with status “not yet recruiting,” “recruiting,” or “active, not recruiting” were sourced from ClinicalTrials.gov (NIH registry).

Therapeutic targets		Drug names	Conditions	Phases	Status	Source	References
Integrin	αvβ5	18F‐FP‐R01‐MG‐F2	Pancreatic carcinoma	Early phase 1	Completed	NCT02683824	[403]
	αvβ3	68Ga‐NODAGA‐E[c(RGDyK)]2	Neuroendocrine tumors	Phase 2	Completed	NCT03271281	[404]
	αvβ3, αvβ5	[18F]Fluciclatide	Ovarian neoplasm	Phase 1	Completed	NCT01608009	[405]
	αvβ3, αvβ5, αvβ6	cRGD‐ZW800‐1	Pancreas adenocarcinoma, cholangiocarcinoma	Phase 2	Completed	NCT05518071	[406]
	β1	OPC 415	Multiple myeloma	Phase 1, phase 2	Completed	NCT04649073	[407]
		131I‐L19SIP	Cancer	Phase 1, phase 2	Completed	NCT01242943	[408, 409]
		OS2966	Glioblastoma	Phase 1	Terminated	NCT04608812	[410]
	α4β1	7HP349	Solid tumors	Phase 1	Completed	NCT04508179	[411]
	α5β1	MINT1526A	Solid cancers	Phase 1	Completed	NCT01139723	[412]
		PF‐04605412	Advanced nonhematologic malignancies	Phase 1	Terminated	NCT00915278	[412]
		Volociximab	Stage IV melanoma	Phase 2	Terminated	NCT00369395	[413]
			Renal cell carcinoma, metastases	Phase 2	Terminated	NCT00100685	[413]
			Ovarian cancer, primary peritoneal cancer	Phase 1, phase 2	Completed	NCT00635193	[414]
			Pancreatic cancer	Phase 2	Completed	NCT00401570	[412]
			Non‐small cell lung cancer (NSCLC)	Phase 1	Completed	NCT00654758	[413, 415]
			Ovarian cancer, peritoneal neoplasms	Phase 2	Completed	NCT00516841	[413]
FAK		Defactinib	Advanced solid tumors, solid tumors, pancreatic cancer	Phase 1	Completed	NCT02546531	[416]
			NSCLC, lung cancer	Phase 2	Completed	NCT01951690	[417]
			Nonhematologic cancers	Phase 1	Completed	NCT01943292	[418]
			Cancer	Phase 1	Completed	NCT00787033	[419]
			Malignant pleural mesothelioma	Phase 2	Terminated	NCT01870609	[420]
		GSK2256098	Intracranial meningioma, recurrent meningioma, NF2 gene mutation	Phase 2	Recruiting	NCT02523014	[421]
			Cancer	Phase 1	Completed	NCT01138033	[422]
		IN10018	Pancreatic cancer	Phase 1, phase 2	Recruiting	NCT05827796	
			Platinum‐resistant ovarian cancer	Phase1, phase 2	Recruiting	NCT05551507	[423]
			Metastatic melanoma	Phase 1	Active, not recruiting	NCT04109456	
			Gastric cancer	Phase 1	Completed	NCT05327231	[424]
ROCK1/2		Fasudil	Ovarian cancer	Phase 2	Recruiting	NCT06890858	
			Prostate CA	Phase 2, phase 3	Not yet recruiting	NCT06861192	
ROCK2		AT13148	Advanced solid tumors	Phase 1	Completed	NCT01585701	[425]
		Belumosudil	Multiple myeloma	Phase 1, phase 2	Recruiting	NCT06105554	
Nonmuscle myosin II		MT110	Solid tumors	Phase 1	Completed	NCT00635596	[426]
		MT125	Glioblastoma	Phase 1	Not yet recruiting	NCT07185880	
YAP		VT3989	Solid tumor, mesothelioma, NSCLC	Phase 1, phase 2	Recruiting	NCT04665206	[427]
		IAG933	Mesothelioma	Phase 1	Active, not recruiting	NCT04857372	[428]
		Verteporfin	Glioblastoma, recurrent glioblastoma	Phase 1, phase 2	Recruiting	NCT04590664	[429]
			Prostate cancer	Phase 1, phase 2	Recruiting	NCT06807359	
			Breast neoplasms	Phase 1, phase 2	Completed	NCT02872064	[430]

Unfortunately, most molecules in mechanotransduction are still at the stage of preclinical tool small molecules, such as these mentioned in Table 5. Besides the most classic molecules in mechanotransduction, inhibitors targeting SEPT9, which can affect actin polymerization status, have recently been reported and can serve as reference [431]. These findings indicate that drug development targeting mechanotransduction, in addition to evaluating the clinical effects of marketed drugs in different diseases, also requires further clarification of the components of intracellular mechanotransduction pathways and their relationship with cellular mechanical states, which would aid in drug design and development.

**TABLE 5:** Drugs affecting mechanical biomarkers.

Mechanical biomarkers	Drug name	Drug type	Effect	References
RhoA	Rhosin	Small molecule	Rhosin binds to the surface area surrounding Trp58 of RhoA, inhibiting GEF‐catalyzed RhoA activation.	[389]
	Y16	Small molecule	Y16 prevents LARG from interacting with RhoA.	[432]
	Clostridium botulinum exoenzyme C3	ADP‐ribosyl transferase	It transfers ADP‐ribose to Rho GTPase, specifically modifying and inactivating it.	[391]
Nonmuscle myosin II	Blebbistatin	Small molecule	They inhibit activity of nonmuscle myosin II.	[402]
	MT228	Small molecule		[433]
Actin	Latrunculin A	Small molecule	They inhibit actin polymerization.	[399]
	Cytochalasin D	Small molecule		[400]
	Jasplakinolide	Small molecule	It induces actin polymerization and stabilizes pre‐existing actin filaments.	[401]
N‐WASP	Wiskostatin	Small molecule	Wiskostatin stabilizes its closed autoinhibitory conformation, preventing the activation of the Arp2/3 complex.	[434]
Arp2/3	CK‐636	Small molecule	They inhibit the Arp2/3 complex to suppress actin polymerization.	[435, 436]
	CK‐666			
	CK‐869			
	Benproperine phosphate	Small molecule	It weakens Arp2/3 function to reduce the rate of actin polymerization.	[437]
Formin	SMIFH2	Small molecule	It inhibits actin polymerization by Formins.	[438]
SEPT9	SEPT9‐IN‐1	Small molecule	It exhibits cytotoxicity, suppression of cell motility and invasion, and disruption of SEPT9–microtubule and SEPT9–actin interactions.	[431]
MRTF	CCG‐1423	Small molecule	They prevent the interaction between MRTF‐A and other RPEL‐family of proteins, decrease nuclear import of MRTF‐A. Also, they inhibiting MICAL‐2 increases nuclear G‐actin and decreases nuclear MRTF‐A.	[393, 394, 395, 396, 397, 439]
	CCG‐203971			
	CCG‐222740			
	CCG‐232601			

## 6 Summary and Prospects

This review systematically summarizes the composition, structure, and characteristics of the cellular microenvironment, explores the mechanisms of mechanotransduction, introduces relevant research techniques and clinical tools, and analyzes the roles of the cellular microenvironment and mechanotransduction in diseases progression, as well as their potential clinical therapeutic targets. The insights provided here offer a comprehensive reference for future research and directions in diagnosis and treatment.

### 6.1 Biological Challenges

This review demonstrates, from the perspective of cell–ECM interactions, that the maintenance of mechanical homeostasis requires cells to establish an intact internal mechanical signaling chain, with a focus on the ligand–integrin–actomyosin–nucleus pathway. Ion channels [440, 441], glycocalyx [442, 443], and other components [444, 445, 446] that can also respond to external mechanical signals have already been summarized and discussed in relevant reviews, so they are not discussed in detail here.

The mechanical equilibrium of cell–ECM corresponds to the mechanical microenvironment experienced by cells in many solid tissues in vivo. However, even more complex mechanical environments exist in the body. The cell–ECM interactions of endothelial cells, which sense different types of forces on the apical and basal surfaces, are influenced not only by the type of substrate but also by FSS on the apical surface. The molecular mechanisms in mechanotransduction pathways may be even more complex. Completely suspended cells, on the other hand, can establish force equilibrium without relying on a substrate. Whether this is due to compensatory roles of other molecules in the mechanotransduction pathways remains unknown, suggesting that cancer cells undergoing distant metastasis may have a stronger compensatory ability for disrupting mechanical homeostasis. At the same time, there are still significant bottlenecks in better simulating the mechanical forces experienced by different parts of the human body in vitro, such as stiffness, shear, tension, and stretching. Taking stiffness as an example, in addition to considering the biocompatibility of the material itself, the mechanical properties of the material cannot be ignored. Factors such as stress stiffening [447] and stress relaxation [448] of polymer materials may have important impacts on the proper formation of cellular internal force structures.

The mechanical identities of biochemical molecules inside cells also need further elucidation. The article only mentions the mechanical markers that have been studied more extensively so far, and there are still many molecules that can directly respond to the force environment whose identities need to be clarified. The cytoplasm envelops the outside of the nucleus, and forces can be transmitted through the cytoskeleton between them or act directly [449] on the nucleus. How various molecules and condensates within the nucleus, which are related to cell fate, respond to changes in force is worth further investigation.

### 6.2 Technological Challenges

At the cellular level, technologies require applying mechanical stimulation to cells and measuring mechanical properties only from outside the biological system. Meanwhile, how to adjust the microenvironment parameters of each technology to make the measured data most consistent with physiological and pathological conditions still needs to be further clarified. Combining multiple detection technologies can complement each other's shortcomings to a certain extent, but how the obtained force spectrum reflects the true mechanical state of the biological system requires continuous theoretical improvement and guidance from mathematical models, involving collaboration among scientists from multiple disciplines.

The prospects for technological development at the molecular level are significant and are directly related to mechanotransduction processes. Existing molecular probes are based on widely accepted biological or mechanical processes, with the most prominent being molecular conformational changes, suggesting that when designing corresponding probes, it is necessary to consider whether the existing forms of the molecules are directly affected by force. An intuitive manifestation of force is the change in intermolecular distance, so the photophysical properties of molecules become an important consideration in probe design. In addition to direct application inside cells, to improve the resolution of molecular mechanical measurements on cell surfaces, the correlation between molecular conformation and magnetism is also considered in material design. However, the design of molecular probes inside cells is affected by the complex biochemical environment, and there is an urgent need to address how to design precise localization while improving imaging resolution and accuracy.

The actual parameters of clinical diagnostic technologies correspond to some extent with data obtained at the cellular level, reflecting the progression of diseases through the overall elastic response of tissues. The precision of technological detection limits the accuracy of clinical disease diagnosis to a certain extent. What urgently needs to be addressed is how to correlate tissue elasticity at the macroscopic level with precise molecular pathogenic mechanisms, which holds promise for achieving precise diagnosis and targeted treatment.

### 6.3 Translational Challenges

Translational challenges mainly focus on the following three aspects: how cellular and molecular mechanisms guide the improvement of clinical diagnostic methods, the role of mechanotransduction in the occurrence and development of diseases, and the the urgent need to expand mechanical biomarkers and targeted drug design.

#### 6.3.1 The Gap Between Molecular Mechanisms and Clinical Applications

Since many mechanisms of mechanotransduction are derived from experiments at the cellular and molecular levels, it is difficult to verify their biological significance, and these experiments often cannot be directly correlated with actual clinical diseases. For example, current clinical diagnostic tools related to mechanical markers rely only on macroscopic tissue elasticity, and the use of molecular‐ and cell‐level detection techniques in pathogenesis is very limited, largely due to a restricted understanding of the relationship between mechanical mechanisms and tissue pathology. Although we can currently only obtain clinical data at the macroscopic level, this indirectly reflects that tissue mechanical homeostasis may have changed during disease progression, suggesting that targeting molecules involved in mechanotransduction could potentially help in halting disease progression.

#### 6.3.2 Incomplete Understanding of the Mechanism

Although oncogenic mutations are necessary drivers of cancer, they are insufficient to promote tumor formation or progression [450]. Currently, much attention in cancer treatment remains focused on oncogenic mutations. However, the success rate of most cancer treatments remains poor, highlighting that focusing solely on genetic mutations is insufficient for treating cancer. Meanwhile, the importance of the mechanical microenvironment, which plays a crucial role in cell–ECM homeostasis, is gradually emerging. This suggests that forces not only influence cancer development and resistance but may also play a significant role in aging‐related chronic diseases. How the accumulation of force variations affects disease progression remains to be further elucidated.

Oncogenesis is a step‐wise acquisition of sustained proliferation, immortality, invasion and immune evasion that converts normal cells into malignant, metastasizing entities [451], whereas cellular senescence can be induced by telomere shortening and different stress, which ultimately transform into cell‐cycle arrest [452]. Oncogene‐, tumor‐suppressor‐loss‐, or therapy‐provoked senescence within cancers termed cancer‐cell autonomous senescence is likewise maintained by p53/p16^INK4a^/p19^ARF^ signaling and reshapes the microenvironment via the SASP [453]. Hence, prosenescence therapies, SASP reprogramming, and senolytic clearance are being developed to restrain tumors while minimizing systemic toxicity [453].

Although oncogenesis and senescence are often viewed as opposite poles of a proliferation‐apoptosis spectrum, biomechanical remodeling of the TME bridges the two states. Senescent CAFs exemplify this link. p16^INK4a^‐driven senescence locks fibroblasts into a long‐lived, low‐proliferative, high‐secretory state that can rapidly switch to a profibrotic, protumoral phenotype under subsequent injury or neoplastic cues [454]. Yet elevated p16 alone is insufficient to define matrix aging, and immune‐mediated clearance of these cells is frequently impaired [454]. Consequently, their precise tumor‐promoting mechanisms remain incompletely resolved.

Aging systemically lowers many tumor‐supportive factors, but local concentrations can rise. In the aging prostate, collagen I loosening, laminin‐332 loss, laminin‐α4/β1 upregulation, accumulation of cleaved TSP1/SPARC, and a shift toward high‐molecular‐weight hyaluronan reconfigure tissue stiffness, porosity, and ligand presentation [455]. SASP from resident senescent cells amplifies these alterations, converting a tumor‐suppressive niche into a permissive one that, despite global antitumor factor decline, enables mutant epithelial cells to proliferate, attract vasculature, and evade immunity, thereby fostering primary tumor progression in elderly hosts [455]. Thus, aging acts as a temporal variable of the mechanical microenvironment that profoundly influences TME evolution.

Senescence is initiated by p53/pRB pathways that initially serve as gatekeepers to block pre‐neoplastic expansion. However, senescent cell accumulation converts these cells into bad neighbors whose SASP impairs tissue repair, incites chronic inflammation, and stimulates the outgrowth of adjacent mutated clones, illustrating the antagonistic pleiotropy of senescence in tumor suppression versus late‐life tumor promotion [456]. When this phenotypic switch occurs within the TME and how matrix mechanics govern the transition remain open questions. Cancer exploits the antiapoptotic and secretory states of surrounding senescent cells to create a favorable microenvironment, whereas fibrosis reflects the uncontrolled manifestation of the same mechanical‐secretory logic at the organ level.

#### 6.3.3 Underexplored Roles of Mechanical Biomarkers

The dysregulation of cancer‐related Rho GTPase activity is mainly caused by the overexpression or imbalance of regulatory factors or effectors. Cancer genome‐wide data have provided substantial evidence of abnormal expression and function of Rho, GEF, GAP, and effectors, but there is a lack of target validation in primary cancer cell lines and animal models, and existing tool molecules mostly remain at the proof‐of‐concept stage, leading to slow development of Rho GTPase‐related drugs [457]. The druggability of actomyosin is primarily limited by the widespread distribution of actin and myosin. Myosin exists in various isoforms and plays important roles in many normal tissues, such as myocardium and smooth muscle. MT110 [433] and MT125 [458] were able to enter clinical trials because of their good targeting specificity to nonmuscle myosin II and superior blood‐brain barrier penetration. Regarding actin, its high intracellular content causes a quantitative challenge for inhibitors to be effective. Therefore, when designing drugs targeting actin, more consideration should be given to the structural characteristics of its assembly in cells and its coupling with the mechanical environment. Inhibitors of N‐WASP, Arp2/3, and Formin can be referenced. In addition, the mechanical roles of many intracellular biochemical molecules remain to be elucidated, and they may exhibit different mechanical sensitivities in different diseases, which is crucial for understanding how old drugs can be repurposed.

### 6.4 Emerging Directions

In terms of mechanotransduction research, it is necessary to identify more biochemical molecules that can be regarded as mechanical biomarkers and to investigate whether these mechanical biomarkers exhibit different functional forms in various diseases. At the same time, it is important to recognize that the exertion of force involves the maintenance of homeostasis, and these mechanical biomarkers should be regarded not merely as biochemical molecules, but rather as active players in the overall force balance. At the same time, more refined and personalized analysis of existing sequencing data is needed to avoid missing important molecules.

From a technical perspective, it is essential to develop more molecular level techniques to analyze forces on a more microscopic scale, achieve integrated application of multiple existing technologies, and develop theoretical models based on current data that better reflect real physiological and pathological conditions to aid in interpreting clinical data.

In terms of treatment, in addition to advancing current clinical trials, it is necessary to develop more preclinical molecular drugs targeting mechanical biomarkers to help understand their relationship with cell–ECM forces. At the same time, attention should be paid to whether existing preclinical drugs can be improved and translated to clinical use, and whether existing clinical drugs may have therapeutic effects in other diseases by altering mechanotransduction pathways.

## Author Contributions

Yicen Long and Peng Wang drafted the manuscript. Peng Wang, Yicen Long, Xiaojing Liu, and Jiacheng Lei prepared the figures. Xiaojing Liu, Qiang Wei, and Baihai Su edited and revised the manuscript. The authors used KIMI for language polishing in the manuscript. The article has received approval from all authors.

## Ethics Statement

The authors have nothing to report.

## Conflicts of Interest

The authors declare no conflicts of interest.

## Data Availability

The authors have nothing to report.
